# A community-wide campaign to promote physical activity in middle-aged and elderly people: a cluster randomized controlled trial

**DOI:** 10.1186/1479-5868-10-44

**Published:** 2013-04-09

**Authors:** Masamitsu Kamada, Jun Kitayuguchi, Shigeru Inoue, Yoshiki Ishikawa, Hiromu Nishiuchi, Shimpei Okada, Kazuhiro Harada, Hiroharu Kamioka, Kuninori Shiwaku

**Affiliations:** 1 Department of Environmental and Preventive Medicine, Shimane University School of Medicine, 89-1 Enya-cho, Izumo, Shimane 693-8501, Japan; 2 Japan Society for the Promotion of Science, 5-3-1 Koujimachi, Chiyoda-ku, Tokyo 102-8471, Japan; 3 Physical Education and Medicine Research Center UNNAN, 1212-3 Mitoya, Mitoya-cho, Unnan, Shimane 690-2404, Japan; 4 Department of Preventive Medicine and Public Health, Tokyo Medical University, 6-1-1 Shinjuku, Shinjuku-ku, Tokyo 160-8402, Japan; 5 Department of Public Health, Jichi Medical School, 3311-1 Yakushiji, Shimotsuke, Tochigi 329-0498, Japan; 6 Data Science Research Institute, 3-10-41 Minami-Aoyama, Minato-ku, Tokyo 107-0062, Japan; 7 Physical Education and Medicine Research Foundation, 6-1 Nunoshita, Tomi, Nagano 389-0402, Japan; 8 Graduate School of Sport Sciences, Waseda University, 2-579-15 Mikajima, Tokorozawa, Saitama 359-1192, Japan; 9 Faculty of Regional Environment Science, Tokyo University of Agriculture, 1-1-1 Sakuragaoka, Setagaya-ku, Tokyo 156-8502, Japan

**Keywords:** Walking, Muscle stretching exercises, Resistance training, Musculoskeletal diseases, Health communication, Social marketing

## Abstract

**Background:**

We aimed to evaluate the effectiveness of a community-wide campaign (CWC) for promoting physical activity in middle-aged and elderly people.

**Methods:**

A cluster randomized controlled trial (RCT) with a community as the unit of randomization was performed using a population-based random-sampled evaluation by self-administered questionnaires in the city of Unnan, Shimane Prefecture, Japan. The evaluation sample included 6000 residents aged 40 to 79 years. We randomly allocated nine communities to the intervention group and three to the control group. The intervention was a CWC from 2009 to 2010 to promote physical activity, and it comprised information, education, and support delivery. The primary outcome was a change in engaging in regular aerobic, flexibility, and/or muscle-strengthening activities evaluated at the individual level.

**Results:**

In total, 4414 residents aged 40–79 years responded to a self-administered questionnaire (73.6% response rate). Awareness of the CWC was 79% in the intervention group. Awareness and knowledge were significantly different between the intervention and control groups, although there were no significant differences in belief and intention. The 1-year CWC did not significantly promote the recommended level of physical activity (adjusted odds ratio: 0.97; 95% confidence interval: 0.84–1.14).

**Conclusions:**

This cluster RCT showed that the CWC did not promote physical activity in 1 year. Significant differences were observed in awareness and knowledge between intervention and control groups as short-term impacts of the campaign.

**Trial registration:**

UMIN-CTR UMIN000002683

## Background

Engaging in regular physical activity (PA) reduces the risks of many chronic diseases [1-5]. However, physical inactivity remains a common public health problem in developed and developing countries [6,7].

PA behaviors are affected by diverse factors at the individual, social, environmental, and policy level [8,9]. Therefore, multilevel and intersectoral approaches seem to be the most successful PA promotion strategies [9,10]. Recently, community-wide interventions involving various campaigns have received broad attention for promoting PA in a wide range of community populations. Such community-wide campaigns (CWC) typically (1) involve many community sectors; (2) include highly visible, broad-based, multi-component strategies; and, (3) may also address other cardiovascular disease risk factors [11,12]. However, well-designed trials assessing the effectiveness of CWC for promoting PA have been lacking [13-22]. A recent review included only one cluster randomized controlled trial (RCT), which focused on adolescents, and concluded that there was a lack of appropriate studies which could show whether this approach is beneficial [22]. The risk of bias, including selection bias in non-randomized studies, in the existing literature might lead to a misunderstanding of effective population strategies. Therefore, it is desirable to conduct randomized studies to obtain more robust knowledge, and advance the body of public health policy and practices.

Focusing on the outcomes targeted by CWCs, there are relatively few studies on flexibility and muscle-strengthening activities compared with those on aerobic activities (e.g., walking) [12-21]. Flexibility and muscle-strengthening activities are generally recommended for older people, and specifically for people with musculoskeletal disorders, as a non-pharmacological treatment [23-27]. Musculoskeletal disorders are a major burden on both individuals and societies [28]. In Japan, musculoskeletal pain has been the most reported subjective symptom [29]. In addition, as arthritis is a potential barrier to PA [30], we cannot ignore the influence of these conditions when promoting PA, especially in older people.

Therefore, this study aimed to evaluate the effectiveness of a CWC for promoting not only aerobic PA, but also flexibility and muscle-strengthening activities in middle-aged and elderly people by conducting a cluster RCT. Our intention was to promote PA through a CWC delivered at the community level. To minimize contamination, the unit of randomization was the community. The hypothesis was that a CWC delivered at the community level would promote engagement in regular aerobic, flexibility, and/or muscle-strengthening activities in middle-aged and elderly people evaluated at the individual level.

## Methods

The COMMUNICATE (COMMUNIty-wide CAmpaign To promote Exercise) study was a cluster randomized controlled, superiority trial, stratified by high, moderate, and low population density, with imbalanced randomization (three interventions; one control). It was conducted in the city of Unnan (population 45364, area 553.7 km^2^), a rural mountainous region in Shimane, Japan. To assess a community level intervention, it is preferable to randomly assign communities rather than individuals to study groups [31]. Full details of the trial protocol can be found in Additional file 1. This study was approved by the research ethics committee of the Physical Education and Medicine Research Center UNNAN.

**Figure 1:**
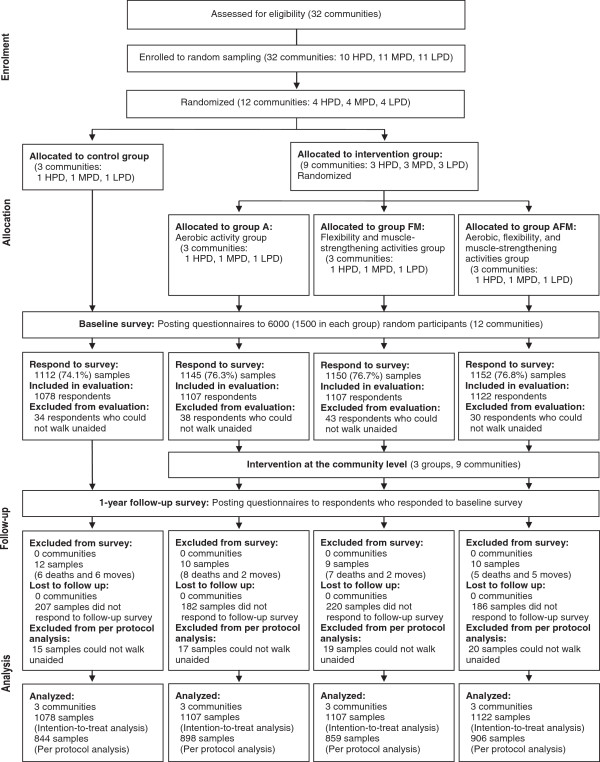
**Flow diagram of the trial process.***Note.* HPD: high population density. MPD: moderate population density. LPD: low population density.

Figure 1 is a flow diagram of the trial process. There are 32 communities within Unnan, with a median population and area of 1292 and 10.8 km^2^, respectively. The eligibility criterion for clusters was all communities in Unnan. The 32 clusters were divided into three groups by population density. Then, 12 clusters were randomly sampled, with stratification by blocking within population density category strata, and randomly allocated to three intervention clusters (i.e., a total of nine clusters) per control cluster (i.e., a total of three clusters). Additionally each cluster in the intervention group was randomly allocated to an aerobic activity group (Group A), a flexibility and muscle-strengthening activities group (Group FM), and an aerobic, flexibility, and muscle-strengthening activities group (Group AFM), each consisting of three clusters. This factorial designed division was for the purpose of subgroup analyses.

Randomization of the clusters was performed by a clerical staff member of Unnan City Hall, blinded to the name and identity of the clusters, using a computer-generated list of random numbers. Another staff member had a list of all cluster names and the relevant numbers and assigned the clusters. Neither staff member was involved in the remainder of this study. We did not conduct any other cluster selection process to minimize the risk of contamination (e.g., geographical distance between individual clusters).

### Intervention

A CWC to promote PA for all middle-aged and elderly (40–79 years) residents living in the communities was conducted as an Unnan City Hall public health project at the cluster level within intervention groups for 1 year (November 2009 to October 2010).

In Group A, mainly walking behavior was promoted for aerobic activity. In Group FM, mainly stretching exercises for back muscles, adductor muscles, gluteus maximus, knee extensor muscles, and knee flexor muscles, and muscle-strengthening activities for trunk flexor, knee extensor, and knee flexor were promoted. These anatomical areas were chosen as key muscle groups for treating low back and knee pain, and the exercises did not require expensive training equipment [25,32,33]. In Group AFM, all of these walking, stretching, and muscle-strengthening activities were promoted.

**Figure 2:**
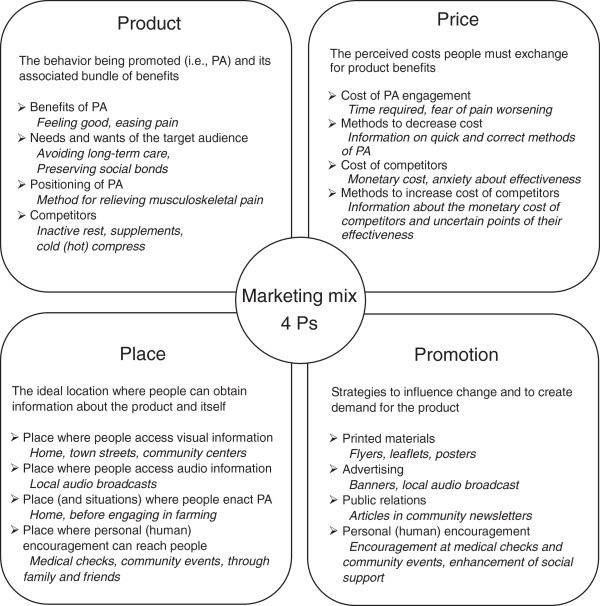
**Concept of marketing mix and example elements of the four Ps for promoting physical activity.***Note.* PA: physical activity

Social marketing applies marketing principles and techniques to create, communicate, and deliver value in order to influence target audience behaviors that benefit society as well as the target audience [34-38]. We adopted the following social marketing processes:

(1) Situational analysis. A situational analysis helps health professionals understand factors that may influence a health campaign and provides background and context to the social marketing plan. We conducted the following situational analyses: background and issue identification, environmental scan, and SWOT (Strengths, Weaknesses, Opportunities, Threats) analysis [37].

(2) Market segmentation and targeting. We used the TARPARE model to determine the primary communication target segment [39]. The model assists the health promotion practitioner to systematically compare and select appropriate target groups when there are a number of segments competing for attention and resources. We placed specific emphasis on the total number of persons in the segment (T), at risk status (AR), and persuasibility (P) as segment priority factors. In the process of market (population) segmentation, we also adopted the stages of change model [40]. However, there were no data regarding the stages of change in PA behavior in this study population, so available behavioral data (i.e., walking times and engagement in stretching) was used to supplement the estimation of segment size. For promotion of aerobic activity, we selected a segment of women, 60–79 years of age, who had an interest in, but were either not engaged in or were insufficiently engaged in, regular walking behavior (less than 150 minutes/week), and who had low back or knee pain, regardless of severity. For flexibility and muscle-strengthening activities, we selected a segment of women, 60–79 years of age, who engaged in flexibility and/or muscle-strengthening activities, either occasionally or daily, and who had low back or knee pain, regardless of severity. The estimated proportion of the target segment of the 40–79-year old population was 19% for aerobic activities and 16% for flexibility and muscle-strengthening activities, based on data from the Shimane Study in Unnan [41]. This targeting did not mean that our CWC ignored PA in non-targeted subjects. Rather, we aimed that the CWC mainly influenced the primary communication target with sophisticated messages and approaches, and then it had a ripple effect on non-targeted subjects.

(3) Setting objectives. The SMART (Specific, Measurable, Achievable, Realistic, Time-based) objective was set for behavioral change as follows: “To increase the percentage of 40–79-year-old individuals who engage in aerobic, flexibility, and/or muscle-strengthening activities in an intervention group from 58% to 66% over a 1-year period.” The baseline percentage was estimated from the available data for Unnan. A previous systematic review reported that the median net increase in the percentage of people who reported being physically active as a result of a CWC was 4.2% (range, -2.9% to 9.4%) [12]. We decided on a target increase of 8% because the area and population sizes were relatively small in this study and the effect of a CWC would be potentially greater within such a community compared with a larger scale CWC (e.g., at state or prefecture level).

(4) Marketing strategy development. A CWC followed the “4 Ps” concept of marketing mix (i.e., making sure the right Product is available at the right Price, in the right Place and is well-Promoted). Figure 2 shows the concept of marketing mix and examples of each component for promoting PA in this study. For example, for Product (i.e., PA), the benefits of the product rather than the product itself were emphasized [38]. Thus, rather than promoting PA *per se*, a CWC should promote ideas such as feeling good, having increased energy or longevity, according to the identified views of the target segments [42]. The 4Ps map was devised according to the results of formative research, including interviews with target and other segments about their lives and values. Additionally, in the process of creating messages and materials, pretesting of materials was performed through interviews with the target segment about their feelings toward, and impressions of, such materials.

After the social marketing process was completed, the key message of “*Be active to cure your low back and knee pain*” (originally in Japanese) was selected and delivered within all intervention groups as the common key message. Cooperative relationships were developed with education and sports organizations, the regional development departments of Unnan City Hall, the Unnan Police Department, each community’s self-administered organization, Senior Citizens’ Club, schools and clinics.

The CWC consisted of three components, namely:

(1) Information delivery. Flyers, leaflets, community newsletters, posters, banners, and local audio broadcasts (samples available in Additional files 2 and 3).

(2) Education delivery. Outreach health education program and mass- and individual encouragement by professionals during medical check-ups and various community events, including sports events and festivals. Mass-encouragement included the delivery of a motivating talk and demonstration of each type of PA using a common procedure to ensure standardization of the intervention and individual encouragement, including short face-to-face promotion and counseling on PA while waiting for medical check-ups, etc.

(3) Support delivery. Development of social (peer) support, i.e., promoting encouragement by community leaders and lay health workers; material support, i.e., arranging for residents to obtain light-reflective material for walking safety, pedometers [43] (Group A and AFM), and videotapes and DVDs on flexibility and muscle-strengthening activities (Group FM and AFM) at each relevant community center; and professional support, i.e., establishing a call center for questions about PA and requests for outreach programs.

The CWC met the definition of a community-wide intervention as set out in Baker and colleagues’ review [22]. It was possible that residents travelled between the different communities for shopping, commuting, seeing a doctor, etc. In order to avoid contamination of the intervention, flyers, leaflets, and community newsletters were delivered to the household directly in the intervention communities, and the audio messages were only delivered to households in the intervention communities by using the cable network (i.e., not radio or terrestrial TV). Educational activities were implemented only at community events in which all participants were residents living in the relevant intervention community. In the control group, public health services were delivered by Unnan City Hall as usual.

### Population-based evaluation

The effectiveness of the intervention was evaluated by a population-based survey, which was a prospective cohort design. As a baseline survey, self-administered questionnaires were mailed to random participants in October 2009. A computer-based resident registry system was used for random sampling. Eligible respondents were all men and women aged 40 to 79 years living in the 12 study communities. Those excluded were individuals in assisted living facilities, those who require long-term care, or those who could not complete the questionnaires themselves due to disability. Based on the information obtained from the questionnaires, those unable to walk unaided were also excluded from the analyses. One-year follow-up questionnaires were mailed to the baseline respondents in October 2010. Respondents confirmed by the registry system as having died or moved were excluded from the follow-up.

All respondents gave written informed consent to participate in these cohort surveys at baseline. The content of the questionnaires was the same for all residents. Both participants and data collectors were randomly sampled residents. Residents and the CWC collaborators (e.g., community self-administered organization staff, Senior Citizens’ Clubs, schools and clinics) were blinded to (not informed about) the study design and hypothesis (i.e., the existence of the control group and cluster allocation) [44]. The implementing staff of the CWC (intervention providers), data analysts, the Mayor, Vice-Mayor, supervisory employees, and public health nurses of Unnan City Hall were not blinded to the cluster allocation.

### Measures

#### Primary outcome

The primary outcome was the change in engagement in regular PA evaluated at the individual level from baseline to 1-year follow-up. If respondents met any one of the following three conditions, they were defined as “engaging in regular PA”: (1) engaging in 150 minutes/week or more of walking, (2) engaging in daily flexibility activity, or (3) engaging in muscle-strengthening activities two or more days/week. The threshold of these conditions was based on the PA recommendations of the American College of Sports Medicine and the American Heart Association [1,23]. We chose this primary outcome, because the intervention promoted specific types of PA rather than comprehensive (all types of) PA. We considered that questions about specific varieties of PA (i.e., walking, flexibility, muscle-strengthening activities) would be more sensitive than the comprehensive PA questionnaires (e.g., the International Physical Activity Questionnaire [45]).

Walking time for both recreation and transport was considered as engagement in walking. Respondents were asked about the number of days per week and the mean number of minutes walked per day, for recreation and transport separately, to give the weekly total minutes of walking time. Frequency of engagement in flexibility activity was assessed categorically (daily, not daily but occasionally, not at all). Walking and flexibility items were adopted from the questionnaire used in the Shimane Study [41]. The 1-week test-retest reliability of the walking questionnaire was acceptable (Spearman’s ρ = 0.79) and has been described elsewhere [41]. The criterion-related validity of this self-administered walking questionnaire compared with average daily step counts recorded by a uniaxial accelerometer (Lifecorder, Suzuken Co., Ltd., Nagoya, Japan [46,47]) was also found to be acceptable (Spearman’s ρ = 0.38) in 95 elderly subjects (40 men and 55 women) aged 74.9 ± 4.5 (range, 62–85) years living in the city of Unnan. The weekly number of days engaged in muscle-strengthening activity was assessed by asking “Do you usually do activities to maintain and/or improve muscles and/or muscle strength (e.g., sit-ups, squats, knee extensions)?” The test-retest reliability of the flexibility and muscle-strengthening activities was assessed by mailing self-administered questionnaires; the questionnaires were mailed twice at an interval of 10 days. The subjects were 500 random participants aged 40–84 years living in Unnan communities other than the 12 study communities of the COMMUNICATE study. Subsequently, the data of 206 individuals (100 men and 106 women) aged 63.4 ± 11.9 (40–84) years were analyzed. The results showed a moderate and acceptable value of weighted kappa (0.72, p < .001) for flexibility and Spearman’s rho (0.75, p < .001) for muscle-strengthening activity.

#### Secondary outcomes

Low back and knee pain were evaluated as secondary outcomes. A visual analog scale (VAS) from 0 mm (no pain) to 100 mm (most intense pain) was used to assess pain intensity [48]. Chronic musculoskeletal pain was defined as current pain lasting longer than 3 months within the past 12 months [49]. These pain outcomes represented possible benefits or harm related to the CWC. The test-retest reliability of the pain questionnaires also showed moderate and acceptable values of Spearman’s rho (low back: 0.70, p < .001; knee: 0.78, p < .001) for VAS scores and Cohen’s kappa (low back: 0.49, p < .001; knee: 0.72, p < .001) for chronic pain in the same population as that for the flexibility and muscle-strengthening activities questionnaires.

**Figure 3:**
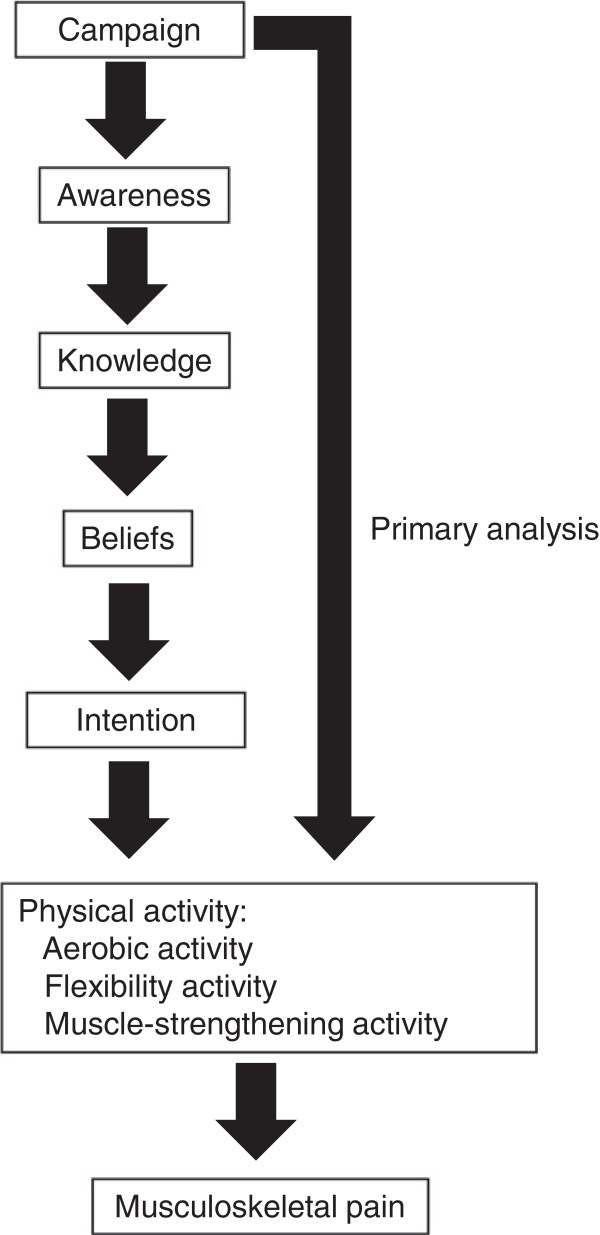
Logic model for community-wide campaign to promote physical activity.

We hypothesized one logic model indicating that change would be induced by the CWC in the following order: awareness; knowledge; belief; intention; and finally PA (Figure 3). This model was adopted from Cavill and Bauman’s model for mass media campaigns [42]. According to our model we evaluated awareness, knowledge, belief, and intention as exploratory analyses. Awareness was evaluated by asking: “Have you seen or heard messages (the campaign) recommending PA, such as “be active to cure your low back and knee pain,” “let’s walk,” or “let’s stretch” (all originally in the Unnan dialect of Japanese) in the last year (November 2009 to October 2010)?” Then, participants answered yes or no to receiving the following CWC components: (1) posters, leaflets, and banners; (2) local audio broadcasts; (3) mass- and individual encouragement by health professionals in community-based medical check-ups and at other community events; (4) individual encouragement by family, friends, and/or neighbors; and (5) advice from physicians and medical staff in medical institutions. Knowledge, belief, and intention were evaluated by asking participants to answer yes or no to the following questions: “Do you know that physical activity is effective for reducing low back and knee pain?”; “Do you think (believe) that physical activity is effective for reducing low back and knee pain?”; and “Do you intend to engage in physical activity within the next 6 months in order to reduce low back and knee pain?” These aspects were only measured at follow-up.

As covariates, we examined sex, age, body mass index (BMI) calculated from self-reported weight and height in kg/m^2^, self-rated health, years of education, employment status, engagement in farming, and chronic disease history, because most of these have been reported to have an impact on PA [8,50-52].

#### Implementation evaluation

We assessed the implementation of the intervention as a process evaluation. For information delivery, the numbers of flyers, leaflets, posters, community newsletters, and banners distributed were recorded. The number of times and the duration of local audio broadcasts were also recorded. For education delivery, a case report form which included the number of attending participants was used, and all health education program and mass- and individual encouragement by professionals were recorded. The quasi-population coverage rate for such educational activities was calculated as gross numbers of participants divided by the population aged 40–79 years in the relevant community. Finally, for support delivery, we recorded the implemented interventions in each community, and these were tabulated and assessed for the degree to which the implementation was adequate for the relevant community.

### Statistical analyses

We calculated our planned sample size of nine clusters and 4500 participants in the intervention group, and three clusters and 1500 participants in the control group (a total of 6000 participants in the 12 clusters) on the assumption of a 50% response rate at baseline to detect an 8% difference in change in engagement in regular PA between the intervention and control groups, taking into account the design effect by cluster randomization [31]. Based on available data in Unnan, the estimated rate of participation in regular PA at baseline was 58% with an estimated intracluster correlation coefficient of 0.00174. We used the chi-square test with imbalanced randomization (three interventions; one control), a two-sided 5% significance level, and a power of 90%.

#### Primary and secondary analyses

We conducted multi-level analyses, taking into account the cluster randomized design. We compared the nine intervention clusters to the three control clusters for the primary outcome of engagement in regular PA at 1-year follow-up using a generalized linear mixed model (GLMM) with sex, age, BMI, self-rated health, years of education, employment, farming, chronic low back and knee pain, chronic disease history, category of population density, engagement in regular PA at baseline, and group allocation (i.e., intervention or control) as fixed effects, and community (cluster) where respondents lived as a random effect.

As secondary analyses, we compared the nine intervention clusters to the three control clusters for changes in VAS pain scores and chronic low back and knee pain using the GLMM, as for the primary analysis. We also compared each intervention group (i.e., Groups A, FM, and AFM) with the control group for primary and secondary outcomes, and changes in engagement in each of the different activities (i.e., aerobic, flexibility, and muscle-strengthening activities) using the same GLMM.

As exploratory analyses, we first analyzed the difference in awareness, knowledge, belief, and intention at follow-up between the intervention and control groups using the GLMM, while controlling for other covariates. Second, in order to assess the logic model for the CWC, we analyzed the associations among variables in the logic model, i.e., awareness, knowledge, belief, intention, and regular PA, in the intervention group using the GLMM and controlling for other covariates. Because there were no baseline data on these variables, these analyses were cross-sectional.

We assigned cluster-specific mean values to missing values for the intention-to-treat analyses, which included all baseline respondents who could walk unaided. When the models did not converge, the analyses were performed using the data of respondents without missing values, i.e., per protocol analyses. For all analyses, we used the forced entry method. Significance was set at p < .05. Analyses were carried out using SAS version 9.1.3 and IBM SPSS Statistics 19.0.

## Results

### Campaign implementation

Table 1 indicates the dose of the implemented information, education, and support delivery in each community. All three components of the CWC were implemented in all intervention communities, although some components were weakly or not implemented in some communities because of the lack of resources and/or the features (e.g., low population) of the relevant community. Information was delivered as follows: leaflets and flyers were distributed to all 4036 households at least twice; posters were hung at 276 sites, including small assembly houses and downtown shops; banners were placed in all community centers; and 60–90 second audio messages were broadcast directly to each household 12 times using a cable network. For education delivery, mass- and individual encouragement activities were conducted by professionals 142 times in total, with a median of 14 times for each community. Approximate gross numbers of participants involved in such activities were 7160 in total, 4610 of whom were 40–79 years old. This covered about 62% of the population aged 40–79 years and living in the intervention communities. Communities with a low population tended to have a quasi-population coverage rate greater than 100%, indicating that some residents received the educational activities multiple times during the intervention period. For support delivery, light-reflective materials were distributed at community events for Group A and AFM; videotapes and DVDs were available for loan at community centers for Group FM and AFM; and a call center was established for all groups.

**Table 1:** Implementation of information, education, and support delivery in communities: COMMUNICATE Study

	**Group A**				**Group FM**				**Group AFM**			
Community	1	2	3	total	4	5	6	total	7	8	9	total
Population density category	HPD	MPD	LPD		HPD	MPD	LPD		HPD	MPD	LPD	
Information delivery												
(Visual information)												
Flyers or leaflets (times distributed to all households)	4	4	4	12	4	2	4	10	3	3	4	10
Posters (numbers hung)	72	17	12	101	28	25	19	72	53	26	23	102
Community newsletters (times articles about CWC appeared)	1	2	4	7	3	0^a^	1	4	2	1	2	5
Banners (numbers placed)	2	2	2	6	2	2	2	6	3	2	2	7
(Audio information)												
Local audio broadcasts (times audio messages broadcasted)	12	12	12	36	12	12	12	36	12	12	12	36
Education delivery												
Times educational activities implemented	20	13	14	47	14	18	11	43	17	21	14	52
Gross numbers of participants (A)	582	330	288	1200	711	768	399	1878	605	473	454	1532
(Population aged 40–79 years (B))	1174	689	269	2132	1165	905	673	2743	1246	1044	328	2618
Quasi-population coverage rate (100*A/B, %)	50	48	107	56	61	85	59	68	49	45	138	59
Support delivery^b^												
(Social support)												
Promoting encouragement by community leaders	yes	yes	no		no	no	no		yes	yes	no	
(Material support)												
Loan and selling of pedometers at community center	yes	yes	no		N/A	N/A	N/A		no	yes	no	
Distribution of light-reflective materials at community center	yes	yes	yes		N/A	N/A	N/A		yes	yes	yes	
Loan of video tapes and DVDs on FM activities at community center	N/A	N/A	N/A		yes	yes	yes		no	yes	yes	
(Professional support)												
Establishment of a call center^c^	yes	yes	yes		yes	yes	yes		yes	yes	yes	

*Note*. Group A = aerobic activity; Group FM = flexibility and muscle-strengthening activities; Group AFM = aerobic, flexibility, and muscle-strengthening activities; HPD = high population density; MPD = moderate population density; LPD = low population density; CWC = community-wide campaign; N/A = not applicable.

^a^There was no community newsletter published by the self-administered organization only in Community 5.

^b^Implemented component is indicated as “yes” and unimplemented component as “no” in each community.

^c^A call center was established in Unnan City Hall for all communities.

The standard public health services in control communities included medical health check-ups, 14 health education classes about general lifestyle and disease prevention (a total of 192 participants), and *ad hoc* health counseling during the intervention period.

### Population-based evaluation

Data from a total of 4414 (73.6%) and 3507 (58.5%) respondents were analyzed in the intention-to-treat and per-protocol manner, respectively (Figure 1). Baseline characteristics of the eligible respondents are presented in Table 2. No significant differences between the control and intervention groups were observed.

**Table 2:** Baseline characteristics of participants randomly selected from communities: COMMUNICATE Study

	**Control**	**Intervention**				**p value**^**a**^
		All	Group A	Group FM	Group AFM	
Cluster						
No. of clusters	3	9	3	3	3	
No. of residents	5235	14721	3700	5553	5468	0.64
No. of residents aged 40–79 years	2917	7493	2132	2743	2618	0.93
Population density, mean ± SD, /km^2^	131 ± 137	273 ± 371	433 ± 641	145 ± 46	240 ± 268	0.52
Evaluation participants (eligible respondents)						
No. of participants (eligible response rate)	1078 (71.9)	3336 (74.1)	1107 (73.8)	1107 (73.8)	1122 (74.8)	0.85
Male	510 (47.3)	1540 (46.2)	522 (47.2)	517 (46.7)	501 (44.7)	0.51
Age, years						
Mean ± SD	61.0 ± 10.6	60.7 ± 10.5	61.2 ± 10.7	60.1 ± 10.4	60.6 ± 10.5	0.29
40-59	471 (43.7)	1514 (45.4)	477 (43.1)	522 (47.2)	515 (45.9)	
60-79	607 (56.3)	1822 (54.6)	630 (56.9)	585 (52.8)	607 (54.1)	
Body mass index, kg/m^2^						
Mean ± SD	22.5 ± 3.2	22.6 ± 3.1	22.8 ± 3.2	22.3 ± 2.9	22.6 ± 3.0	0.68
<18.5	83 (8.1)	226 (7.0)	62 (5.9)	88 (8.2)	76 (6.9)	
≥18.5- < 25	744 (72.2)	2352 (72.9)	770 (72.8)	804 (74.8)	778 (71.1)	
≥25	204 (19.8)	650 (20.1)	226 (21.4)	183 (17.0)	241 (22.0)	
Self-rated health						
Excellent/good	878 (81.9)	2722 (82.7)	885 (80.8)	902 (83.0)	935 (84.3)	0.20
Fair/poor	194 (18.1)	569 (17.3)	210 (19.2)	185 (17.0)	174 (15.7)	
Years of education, mean ± SD	11.5 ± 2.3	11.5 ± 2.4	11.5 ± 2.4	11.4 ± 2.3	11.5 ± 2.5	0.72
Employed	695 (69.6)	2101 (68.7)	665 (64.6)	711 (70.0)	725 (71.6)	0.58
Engagement in farming	552 (52.4)	1626 (49.7)	466 (42.7)	627 (58.2)	533 (48.4)	0.13
Chronic disease history^b^	659 (61.1)	2059 (61.7)	679 (61.3)	673 (60.8)	707 (63.0)	0.73
Regular physical activity^c^	574 (64.6)	1745 (63.0)	614 (66.6)	526 (58.3)	605 (64.0)	0.40
Total walking time, mins/week						
Median (interquartile range)	60 (0–210)	60 (0–200)	80 (0–210)	60 (0–180)	60 (0–200)	0.53
≥150	311 (37.7)	914 (36.4)	319 (38.1)	282 (34.1)	313 (37.0)	
Flexibility activity						
Daily	253 (24.4)	772 (23.8)	276 (25.9)	214 (19.8)	282 (25.8)	0.45
Not daily but occasionally	463 (44.7)	1548 (47.7)	524 (49.1)	518 (47.9)	506 (46.3)	
Not at all	320 (30.9)	922 (28.4)	267 (25.0)	349 (32.3)	306 (28.0)	
Muscle-strengthening activity, days/week						
Median (interquartile range)	0 (0–3)	0 (0–3)	1 (0–3)	0 (0–3)	0 (0–3)	0.99
≥2	348 (38.0)	1080 (37.7)	390 (40.9)	310 (33.0)	380 (39.2)	
Median (interquartile range) VAS pain score						
Low back	5 (0–32)	8 (0–36)	8 (0–36)	9 (0–37)	7 (0–32)	0.11
Knee	0 (0–7)	0 (0–13)	0 (0–15)	0 (0–11)	0 (0–12)	0.067
Chronic musculoskeletal pain^d^						
Low back	133 (13.1)	441 (14.1)	145 (13.9)	150 (14.5)	146 (13.8)	0.43
Knee	95 (9.1)	360 (11.2)	115 (10.8)	122 (11.4)	123 (11.4)	0.062

*Note.* Group A = aerobic activity; Group FM = flexibility and muscle-strengthening activities; Group AFM = aerobic, flexibility, and muscle-strengthening activities. VAS = visual analog scale. Figures are numbers (percentages) unless stated otherwise. Sample sizes (denominators) vary due to missing values.

^a^Comparison between control and intervention groups using the chi-square test for binary variables and Mann–Whitney *U*-test for categorical and continuous variables with non-normal distribution.

^b^Having the following disease history: hypertension, hyperlipidemia, diabetes, hyperuricemia, cerebrovascular disease, heart disease, kidney and urologic diseases, liver disease, gastrointestinal disease, endocrine disease, cancer.

^c^Engagement in regular aerobic, flexibility, and/or muscle-strengthening activities. If respondents met any one of the following three conditions, the respondents were defined as “engaging in regular physical activity”: (1) engaging in 150 mins/week or more of walking, (2) engaging in daily flexibility activity, or (3) engaging 2 or more days/week in muscle-strengthening activities.

^d^Current pain lasting longer than 3 months within the past 12 months.

### Primary and secondary analyses

The proportion of respondents who engaged in regular PA decreased from 64.6% to 60.3% in the control group and from 63.9% to 58.7% in the intervention group in the intervention year. The effect size (adjusted odds ratio, OR) of the CWC was not significant (0.97; 95% confidence interval (CI): 0.84–1.14). There were also no significant changes in pain outcomes between control and intervention groups (Table 3). When comparing each intervention group (i.e., Group A, FM, and AFM) with the control group for regular PA, pain outcomes, and each different activity, no significant changes were observed.

**Table 3:** Changes in physical activity and musculoskeletal pain from baseline to 1-year follow-up: COMMUNICATE Study

	**Control (n = 1078)**	**Intervention**								**Intracluster correlation coefficient**^**b**^
		**All (n = 3336)**		**Group A (n = 1107)**		**Group FM (n = 1107)**		**Group AFM (n = 1122)**		
	**No (%)**	**No (%)**	**Effect size**^**a**^	**No (%)**	**Effect size**^**a**^	**No (%)**	**Effect size**^**a**^	**No (%)**	**Effect size**^**a**^	
			**(95%CI)**		**(95%CI)**		**(95%CI)**		**(95%CI)**	
Regular physical activity^c^										
Engaging at follow-up	451 (60.3)	1400 (58.7)	0.97 (0.84-1.14)	482 (60.3)	1.02 (0.84-1.23)	429 (55.9)	0.94 (0.77-1.14)	489 (60.0)	0.97 (0.80-1.17)	0.0014
Change from not engaging to engaging^d^	58 (26.9)	196 (27.3)		59 (27.6)		63 (23.9)		74 (30.7)		
Specific physical activity										
Total walking time, mins/week										
Median (IQR) change	0 (−60-45)			0 (−60-40)	11.1^e^ (−7.02-29.3)			0 (−45-40)	−13.4^e^ (−29.9-3.13)	0.0012
≥150 at follow-up	232 (34.3)			264 (35.4)				252 (34.0)		
Change from not ≥150 to ≥150^d^	66 (18.9)			63 (17.3)				66 (17.1)		
Flexibility activity										
Daily at follow-up	190 (22.9)					167 (19.6)	0.95 (0.75-1.19)	208 (23.2)	1.44 (0.59-3.53)	0.0047
Change from not daily to daily^d^	69 (11.6)					65 (9.8)		70 (11.0)		
Muscle-strengthening activity, days/week										
Median (IQR) change	0 (0–0)					0 (0–0)	−0.14^e^ (−0.30-0.02)	0 (−1-0)	0.24^e^ (−0.15-0.64)	0.0081
≥2 at follow-up	261 (32.5)					226 (27.5)		314 (36.3)		
Change from not ≥2 to ≥2^d^	52 (12.8)					60 (12.6)		86 (19.2)		
VAS pain score										
Median (IQR) change in low back pain	0 (−8-4)	0 (−10-4)	0.66^e^ (−0.63-1.95)	0 (−11-4)	1.53^e^ (−0.62-3.69)	0 (−10-4)	0.54^e^ (−1.03-2.11)	0 (−8-5)	0.59^e^ (−0.98-2.17)	<0.0001
Median (IQR) change in knee pain	0 (0–0)	0 (−1-0)	0.49^e^ (−0.61-1.59)	0 (−1-0)	0.81^e^ (−0.57-2.19)	0 (−1-0)	−0.15^e^ (−1.77-1.47)	0 (−1-0)	0.37^e^ (−0.98-1.72)	<0.0001
Chronic musculoskeletal pain										
Low back pain at follow-up	125 (15.1)	378 (14.5)	0.92 (0.74-1.14)	125 (14.2)	0.91 (0.66-1.25)	128 (15.2)	1.04^f^ (0.63-1.72)	125 (14.1)	1.05^f^ (0.60-1.84)	<0.0001
New incidence of low back pain in 1 year	48 (7.0)	144 (6.8)		49 (6.8)		51 (7.6)		44 (6.0)		
Knee pain at follow-up	81 (9.9)	313 (12.1)	1.20 (0.93-1.54)	106 (12.2)	1.23 (0.91-1.66)	92 (11.0)	1.00 (0.73-1.38)	115 (13.0)	1.25 (0.93-1.70)	<0.0001
New incidence of knee pain in 1 year	28 (3.9)	121 (5.4)		43 (5.7)		35 (4.8)		43 (5.6)		

*Note.* Group A = aerobic activity; Group FM = flexibility and muscle-strengthening activities; Group AFM = aerobic, flexibility, and muscle-strengthening activities. CI = confidence interval; VAS = visual analog scale; IQR = interquartile range. Sample sizes (denominators) of number counts vary due to missing values, although the effect sizes were calculated by the intention-to-treat analyses.

^a^Effect size estimates adjusted for sex, age, body mass index, self-rated health, years of education, employment status, engagement in farming, (chronic low back and knee pain for the analyses of physical activity outcomes), chronic disease history, category of population density of each cluster, and outcome variable at baseline, and clustering effects. Effect size are adjusted odds ratios unless stated otherwise. Larger than one means that the intervention had a positive effect (favorable for physical activity and not favorable for pain).

^b^Intracluster correlation coefficient (ICC) of each outcome variable at follow-up was calculated by using per-protocol samples without imputation as follows: ICC = (BMS - WMS)/(BMS + [K - 1] WMS), where BMS is the between-cluster mean square, WMS is the within-cluster mean square, and K is the average number of respondents per cluster.

^c^Engagement in regular aerobic, flexibility, and/or muscle-strengthening activities. If respondents met any one of three following conditions, the respondents are defined as “engaging in regular physical activity”: (1) engaging in 150 mins/week or more of walking, (2) engaging in daily flexibility activity, or (3) engaging 2 or more days/week in muscle-strengthening activities.

^d^Change from baseline to follow-up.

^e^Effect size is adjusted difference (linear regression coefficient) for continuous variable. Larger than zero means that the intervention had positive effect (favorable for physical activity and not favorable for pain).

^f^Result from per protocol analysis. The model did not converge in the intention-to-treat analysis.

### Exploratory analyses

At follow-up, 79.3% of the respondents in the intervention group were aware of at least one component of the CWC (Table 4). However, 58.7% were also aware in the control group. Significant differences were observed in awareness and knowledge between the control and intervention groups, whereas belief and intention were not significantly different. In the intervention group, all variables in the logic model were significantly associated each other, except for awareness and belief (OR = 1.20; 95% CI 0.97–1.48) (Table 5). The variable with the largest effect size on regular PA was intention (OR = 2.29; 95% CI: 1.85–2.83).

**Table 4:** Awareness, knowledge, belief, and intention in control and intervention communities at 1-year follow-up: COMMUNICATE Study

	**Control (n = 1078)**	**Intervention (n = 3336)**	**Intracluster correlation coefficient**^**a**^	**Adjusted odds ratio**^**b**^
	**No (%)**	**No (%)**		**(95% CI)**
Awareness of campaign:				
Any	471 (58.7)	2044 (79.3)	0.048	2.70*** (2.02-3.58)
Visual information	253 (31.4)	1502 (58.4)	0.071	3.21*** (2.07-4.95)
Audio information	293 (37.6)	1336 (53.4)	0.035	2.08*** (1.36-3.18)
Encouragement (education)	271 (34.6)	1327 (52.5)	0.062	2.30** (1.33-3.97)
Peer support	229 (29.2)	956 (37.7)	0.024	1.50 (0.96-2.34)
Advice from physicians	177 (22.7)	645 (25.5)	0.010	1.21^c^ (0.87-1.67)
Knowledge about physical activity benefit	689 (84.7)	2264 (88.2)	0.0038	1.51^c^* (1.01-2.25)
Belief about physical activity benefit	489 (60.4)	1648 (64.1)	0.0033	0.84^c^ (0.64-1.10)
Intention to engage in physical activity	599 (74.9)	2018 (79.5)	0.0046	1.31 (1.00-1.72)

*Note.* CI = confidence interval. Sample sizes (denominators) of number counts vary due to missing values, although the adjusted odds ratios are calculated by the intention-to-treat analyses.

^a^Intracluster correlation coefficient (ICC) of each outcome variable at follow-up was calculated by using per-protocol samples without imputation as follows: ICC = (BMS - WMS)/(BMS + [K - 1] WMS), where BMS is the between-cluster mean square, WMS is the within-cluster mean square, and K is the average number of respondents per cluster.

^b^Odds ratio >1 means that more people in intervention group than control group answered yes to the question. Adjusted for sex, age, body mass index, self-rated health, years of education, employment status, engagement in farming, chronic low back and knee pain, chronic disease history, category of population density of each cluster, and engagement in regular physical activity at baseline, and clustering effects.

^c^Result from per protocol analysis. The model did not converge in the intention-to-treat analysis.

*p < .05, **p < .01, ***p < .001.

**Table 5:** Associations among variables in the logic model of the community-wide campaign: COMMUNICATE Study

	**Dependent variables**			
	**Knowledge**	**Belief**	**Intention**	**Behavior (regular PA)**
	**OR (95% CI)**	**OR (95% CI)**	**OR (95% CI)**	**OR (95% CI)**
Independent variables				
Awareness	2.70*** (2.08-3.50)	1.20 (0.97-1.48)	1.91*** (1.53-2.39)	1.48*** (1.20-1.82)
Knowledge	-	13.1^a^*** (8.58-20.00)	3.16*** (2.43-4.10)	1.57*** (1.21-2.04)
Belief		-	2.28*** (1.86-2.79)	1.28*** (1.06-1.53)
Intention			-	2.29*** (1.85-2.83)

*Note.* OR = odds ratio; CI = confidence interval; PA = physical activity. Odds ratios are adjusted by sex, age, body mass index, self-rated health, years of education, employment status, engagement in farming, chronic low back and knee pain, chronic disease history, category of population density of each cluster, and engagement in regular physical activity at baseline, and clustering effects in generalized linear mixed model.

^a^Result from per protocol analysis. The model did not converge in the intention-to-treat analysis.

***p < .001.

## Discussion

The present study showed that the 1-year CWC did not promote PA in middle-aged and elderly people. To our knowledge, this is the first study that examined the effectiveness of a CWC for promoting PA in middle-aged and elderly people using a randomized design.

Our results regarding the hypothesized logic model suggest that changes in awareness and knowledge could be observed as short-term impacts of the CWC. Baker and his colleagues’ model also proposed that awareness and knowledge change as short-term impacts, and changes in belief, intention, and PA level are medium-term results in community-wide interventions [22]. It was unknown how long a CWC should be conducted to promote PA. The most frequent duration of interventions in the 25 studies in a recent Cochrane Systematic Review article for community-wide interventions was 1 year (six studies) and the median duration was 3 years with a range of 1–7 years [22]. Our results suggest that 1 year should be considered short-term and that more time is needed for CWCs to attain behavior (PA) change in middle-aged and elderly people.

We used cluster-specific mean values, which might increase the risk of regression to the mean. Reduced variance within each cluster increases the statistical power (increased type 1 error). However, our primary and secondary analyses indicated that the differences between intervention and control group were insignificant even in this situation. Thus, our conclusion about the insignificant difference is considered to be valid.

Significant associations among variables in our hypothesized logic model suggest that this model is effective for process evaluation of a CWC. Respondents who were aware of the campaign were more likely to engage in the recommended level of PA after the campaign than those who were not aware of the campaign. Awareness of peer support and advice from physicians was low (37.7% and 25.5%, respectively). Additional emphasis on peer support and advice from physicians and, if possible, some environmental changes might be effective in a future CWC [8,12,53]. Some voluntary activities (e.g., monthly walking events in two communities in Group A, and stretching exercises in a cultural, arts and recreational group in one community in Group AFM) started after the 1-year campaign finished. Therefore, intervention effects could be observed as late behavior change. In response to the results of the current study, campaign improvement and maintenance had been implemented and a 3-year follow-up evaluation planned. The CWC strategy has to focus on changing belief [54], intention, and the actual behavior of the population in this next phase.

We assessed the implementation process by using the output data. Most parts of the three components of the CWC were implemented in all intervention communities, although some components were weakly or not implemented in some communities. An effort to deliver intervention components equally at the same level of dose across all communities is an essential part of this kind of community intervention trial. In addition, in order to assess the external validity (generalizability) of this public health intervention, further analysis using the RE-AIM framework will also be of value [55]. For example, the data on the (quasi-)population coverage rate for education delivery, as indicated in this study, will be valuable data for the “Reach” component of RE-AIM and for understanding how much intervention is necessary to achieve population-level behavioral change.

We did not observe any significant changes in pain outcomes. This is not unexpected, as pain improvement generally occurs after an increase in PA level. No significant change in pain might also indicate that no harm was observed.

In this study, no differences were observed when comparing Groups A, FM, and AFM with the control group. Walking is the most common PA of adults and the elderly [56,57]. Flexibility and muscle-strengthening activities can easily be performed at home without special equipment. However, a lack of specific knowledge of how to perform such activities may be a barrier to participation [58]. In Group AFM, the amount of information delivered was greater than that for Group A and FM, thus the burden felt by the former might be greater than by the latter. However, if the CWC could succeed in motivating people to perform all these PAs, then the achieved health benefit would be the greatest [1,23]. In order to develop strategies to disseminate PA recommendations, further studies are necessary to examine whether information about a variety of PAs (e.g., aerobic, flexibility, and muscle-strengthening activities together) can be successfully delivered in community-wide interventions and whether differences exist by type of PA.

This study had some limitations. First, a self-administered questionnaire, which might be subject to recall bias, was used for outcome measurement. In smaller-scale clinical trials, objective measures (e.g., devices to assess movement directly) can be used as indices of change. However, in broader-reach trials, objective measures are often prohibitively expensive, burdensome to participants and logistically difficult. Thus, in broad-reach trials, self-report measures frequently must be relied on. Brief self-report measures have been suggested as useful for their comparability of population PA estimates and have low respondent burden [59]. In addition, little is known to date about objective methods to assess daily flexibility and muscle-strengthening activities in population-wide studies. A strength of this study is that we used questionnaires, whose reliability was assessed, to measure these activities. Although the term “usually” could be interpreted in a variety of ways by different participants, the test-retest reliability of the muscle-strengthening activity was moderate and acceptable.

Second, since the awareness of the CWC was extraordinarily high in the control group, it is possible that contamination of the intervention occurred. The proximity of study communities is a possible explanation. Of all the intervention components, the exposure to campaign posters and word-of-mouth from an acquaintance and friends were possible sources of contamination, although efforts to avoid contamination were made when delivering visual and audio information and educational activities. The questionnaire might also have elicited information about general exposure to PA information, rather than just exposure to the CWC. In order to confirm this, we conducted a telephone survey in randomly selected respondents (n = 60) of the 3-year evaluation (the succeeding survey of this study) in December 2012. Results indicated that only 48% (0% for audio information delivery, 7% for mass encouragement) of the respondents who answered that they were aware of the visual information delivery of the campaign in the questionnaire correctly remembered the specific features of the campaign in the control group, compared with 85% (48% for audio information delivery, 62% for mass encouragement) in the intervention group (Chi square test: *p* = 0.012 for visual information, *p* < .01 for audio information, *p* < .01 for mass encouragement). Most of the respondents in the control group tended to answer “yes” to the awareness question because they thought of exposures to PA-related information (e.g., promotion of exercise classes) irrelevant to the campaign. Considering the low percentages (0% and 7%) of the questions answered correctly, contamination of audio broadcast and mass encouragement did not seem to occur broadly whereas visual information delivery (e.g., posters) could be seen even by the residents in the control group. Although the absolute proportion of awareness should be interpreted with caution, it is still clear that the CWC did reach many residents in the intervention communities through the various interventions (e.g., about 62% of the population by education delivery).

Third, single items and “yes”/”no” responses were used for the logic model measures due to space limitation in the questionnaire. They could not capture the depth of knowledge, strength of belief, and/or intention. The items for knowledge and belief also seem to be leading, and it is possible that these items led to response bias. A questionnaire space limitation problem often occurs in large scale community intervention studies. A high volume questionnaire risks lowering the response rate [60]. Short and reliable psychometric items are important to evaluate the psychological factors. In addition, as these analyses on the logic model measures were cross-sectional, we cannot make conclusions about causality.

Regarding the research design of the health promotion studies, although there are some criticisms of RCTs [61], Rosen et al. argued that many of these objections can be eliminated through a better grasp of the basics of RCTs and their proper implementation, and with a better understanding of research ethics in general [62]. A cluster RCT is considered as the preferable study design for this type of community intervention study and it is definitely an important strength of our study. The high response rate and detection of harm from the intervention using a pain questionnaire, which is not usually considered in PA interventions, are also strengths of this study.

## Conclusions

The results of this cluster RCT indicate that the CWC in Unnan, Japan did not promote PA in middle-aged and elderly people in 1 year. Significant differences were observed in awareness and knowledge between intervention and control groups as short-term impacts of the campaign. Our study, with its robust design and excellent community-wide coverage provides valuable information about the time required for a CWC to attain actual behavioral change. Furthermore, the logic model is useful for public health professionals involved in developing, implementing, and evaluating CWCs to promote PA.

## Abbreviations

PA: Physical activity; CWC: Community-wide campaign; RCT: Randomized controlled trial; Group A: Aerobic activity group; Group FM: Flexibility and muscle-strengthening activities group; Group AFM: Aerobic, flexibility, and muscle-strengthening activities group; VAS: Visual analog scale; BMI: Body mass index; GLMM: Generalized linear mixed model; OR: Odds ratio; CI: Confidence interval.

## Competing interests

The authors declare that they have no competing interests.

## Authors’ contributions

MK conceptualized and designed the study, supervised all aspects of its implementation, performed the data analysis, and wrote the brief. JK assisted with the intervention and data collection and analysis. YI, KH, SO, and HK helped plan the intervention and evaluation of the campaign and oversee their implementation. HN served as the biostatistician and performed the data analysis. SI and KS collaborated on all aspects of the study and provided scientific review of the study. All the authors interpreted the findings and reviewed the drafts of the article. All authors read and approved the final manuscript.

## Authors’ information

At the time of the study, MK was also with the Physical Education and Medicine Research Center UNNAN, Unnan, Shimane, Japan.

## Supplementary Material

Additional file 1 **Study protocol.** Study protocol of the COMMUNICATE Study. Click here for file

Additional file 2 **Poster.** Sample material (poster) of the community-wide campaign: COMMUNICATE Study. Click here for file

Additional file 3 **Banner.** Sample material (banner) of the community-wide campaign: COMMUNICATE Study. Click here for file
