# Supplementary material for: A community-wide campaign to promote physical activity in middle-aged and elderly people: a cluster randomized controlled trial
Source: Int J Behav Nutr Phys Act. 2013 Apr 9;10:44. doi: 10.1186/1479-5868-10-44 (PMC3637495; doi:10.1186/1479-5868-10-44)
Supplement: Additional file 1 — Study protocol. Study protocol of the COMMUNICATE Study. [file 1479-5868-10-44-S1.pdf]

Study Protocol

A community-wide campaign to promote aerobic, flexibility, and muscle-strengthening activities in middle- and old-aged people: a cluster randomized controlled trial (COMMUNICATE study).

Trial registration: UMIN-CTR, *UMIN000002683*

Prepared by Physical Education and Medicine Research Center UNNAN and collaborators

Ver. 2.0 Sep. 2010

Ver. 1.0 Oct. 2009, Japanese original version.

Ver. 2.0 Sep. 2010, Translated into English and details described.

COMMUNICATE STUDY PROTOCOL

Table of Contents

1. General information and executive summary……………………..…………..1

2. Introduction……………………………………………………..……………3

3. Design and study location…………………………………..……………...4

4. Intervention…………………………………………..……………………7

4.1. Social marketing……………………………………..……………………7

4.2. Campaign component and implementation…………….…………………11

5. Population-based evaluation……………………………………………………13

6. Measures……………………………………………………………………14

6.1. Primary outcome………………………………………………………14

6.2. Secondary outcomes……………………………………………………14

7. Statistical analyses………………………………………………………15

7.1. Sample size…………………………………………………………15

7.2. Primary and secondary analyses…………………………………………16

8. Data management…………………………………………………………17

9. Ethical considerations, safety, and dissemination……………………………18

10. Financial matters…….……………………………………………………19

11. References…………………………………………………………………20

12. Appendix: Questionnaire (in Japanese)

1. General information and executive summary

This study is to be implemented as one of the public health projects of Unnan City Hall. Head office is in the Physical Education and Medicine Research Center UNNAN, Unnan, Shimane, Japan.

Correspondence:
Physical Education and Medicine Research Center UNNAN
1212-3 Mitoya, Unnan, Shimane 690-2404, Japan.
Phone: +81-854-45-0300, Fax: +81-854-45-5266

E-mail address: kamada@gakushikai.jp (Masamitsu Kamada)

Main researchers and collaborators (study project members):
Masamitsu Kamada,[a,b] Jun Kitayuguchi,[a] Shigeru Inoue,[c] Yoshiki Ishikawa,[d] Hiromu Nishiuchi,[e] Shimpei Okada,[f] Kazuhiro Harada,[g,h] Hiroharu Kamioka,[i] Kuninori Shiwaku[b]

Institutional affiliations:
[a]*Physical Education and Medicine Research Center UNNAN, Unnan City, Shimane, Japan*
[b]*Department of Environmental and Preventive Medicine, Shimane University School of Medicine, Izumo City, Shimane, Japan*
[c]*Department of Preventive Medicine and Public Health, Tokyo Medical University, Shinjuku-ku, Tokyo, Japan*
[d]*Department of Public Health, Jichi Medical School, Shimotsuke City, Tochigi, Japan*
[e]*Health Communication Core, Population Sciences, Medical Oncology, Dana-Farber/Harvard Cancer Center, Boston, MA, USA*
[f]*Physical Education and Medicine Research Foundation, Tomi City, Nagano, Japan*
[g]*Graduate School of Sport Sciences, Waseda University, Tokorozawa City, Saitama, Japan*
[h]*Japan Society for the Promotion of Science, Chiyoda-ku, Tokyo, Japan*
[i]*Faculty of Regional Environment Science, Tokyo University of Agriculture, Setagaya-ku, Tokyo, Japan*

Executive summary

Well-designed trials assessing the effectiveness of community-wide campaigns (CWC) for promoting physical activity are lacking. This study aims to evaluate the effectiveness of CWCs for promoting physical activity. A cluster randomized controlled trial with a community as the unit of randomization will be undertaken using a population-based random-sample evaluation. Self-administered questionnaires will be used to obtain data from 40- to 79 -year-old people living in communities in the city of Unnan, Shimane, Japan. Both participants and data collectors will be randomly-sampled residents and blinded to the study hypothesis. The intervention will be a one-year CWC comprised of aerobic activity (Group A), flexibility and muscle-strengthening activities (Group FM), or all three (Group AFM), and will include information, education, and support delivery, according to a social marketing process. Using computer-generated randomization, we randomly allocated nine communities into intervention groups and three into an evaluation-only control group as clusters. The primary outcome will be change in the engagement in regular aerobic, flexibility, and/or muscle-strengthening activities at one-year follow-up. Secondary outcomes will include low back pain and knee pain.

Trial registration: UMIN-CTR, UMIN000002683

Keywords: walking, muscle stretching exercises, resistance training, musculoskeletal diseases, health communication, social marketing

2. Introduction

Engaging in regular physical activity reduces the risks of many chronic diseases.[1-3] However, physical inactivity remains a common public health problem in developed and developing countries.[4, 5]

Recently, community-wide interventions involving various campaigns have received broad attention for promoting health behaviors in a wide range of community populations. Such community-wide campaigns (CWCs) to increase physical activity are defined as interventions that (1) involve many community sectors; (2) include highly visible, broad-based, multicomponent strategies; and, (3) may also address other cardiovascular disease risk factors. They are also recommended as community preventive services.[6, 7] Well-designed trials to assess the effectiveness of CWC for promoting physical activity have been lacking.[8-15] Implementation of a cluster randomized controlled trial and other well-designed studies, even with practical limitations, would advance the body knowledge of public health policy and practices.

With regard to the content (types of physical activities) of CWC, there are relatively few studies on flexibility (e.g., stretching exercises) and muscle-strengthening activities (e.g., squats) compared to those on aerobic activities (e.g., brisk walking).[7-15] However, flexibility and muscle-strengthening activities are recommended for musculoskeletal disorders like low back pain and osteoarthritis as a non-pharmacological treatment (i.e., exercise therapy).[16-19] Musculoskeletal disorders are a major burden on individuals, health systems, and social care systems, with indirect costs being predominant.[20] In Japan, musculoskeletal pain has been the most reported subjective symptom.[21] In addition, as arthritis is considered to be a potential barrier to physical activity among adults with heart disease,[22] we cannot ignore the influence of these conditions when promoting physical activity, especially in older people. Flexibility and muscle-strengthening activities specifically have been recommended generally for elderly people, not only for those with musculoskeletal disorders, by the American College of Sports Medicine and the American Heart Association.[23]

This study aims to evaluate the effectiveness of a CWC for promoting physical activity, including not only aerobic, but also flexibility and muscle-strengthening activities in middle-aged and elderly people by a cluster randomized controlled trial. Our intention is to promote physical activity through

a CWC delivered at the community level and to minimize contamination the unit of randomization is the community. The hypothesis is that a CWC delivered at the community level promotes engagement in regular aerobic, flexibility, and/or muscle-strengthening activities in middle-aged and elderly people evaluated at the individual level.

3. Design and study location

This study is named the COMMUNICATE (*COMMUNIty-wide CAmpaign To promote Exercise*) study. It is a stratified (high, moderate, and low population density, with imbalanced randomization [3 intervention; 1 control]), cluster randomized controlled, superiority trial in Unnan (population 45,364, area 553.7 km^2), Shimane, Japan. We identified communities as potential study cluster based on that community being characterized by a self-administered organization and historical regional banding. There are 32 such communities within Unnan, that are similar to the public elementary school districts, with a median population and area of 1,292 and 10.8 km^2, respectively. The eligibility criterion for clusters was all communities in Unnan. The 32 clusters were divided into three groups by population density (i.e., 10 high population density (HPD) clusters; 11 moderate population density (MPD) clusters; 11 low population density (LPD) clusters). Then, 12 clusters were randomly sampled with stratification by blocking within population density category strata and randomly allocated to three intervention clusters (i.e., a total of nine clusters: three HPD; three MPD; three LPD) per each control cluster (i.e., a total of three clusters: one HPD; one MPD; one LPD). Additionally, each cluster in an intervention group was randomly allocated in a similar fashion to an aerobic activity group (Group A), a flexibility and muscle-strengthening activities group (Group FM), and an aerobic, flexibility, and muscle-strengthening activities group (Group AFM), each consisting of three clusters (one HPD; one MPD; one LPD). This factorial designed division is for purposes of subgroup analyses. Primary analysis is to be conducted on the parallel-group designed comparison between the nine intervention clusters and the three evaluation-only control clusters. Randomization of the clusters was done using a computer-generated list of random numbers by a clerical staff member of Unnan City Hall, blind to the name and identity of the clusters. Another staff member had a list of all cluster names and the relevant numbers and assigned the clusters. Neither staff member will

be involved in the intervention, evaluation, and analysis of this study.

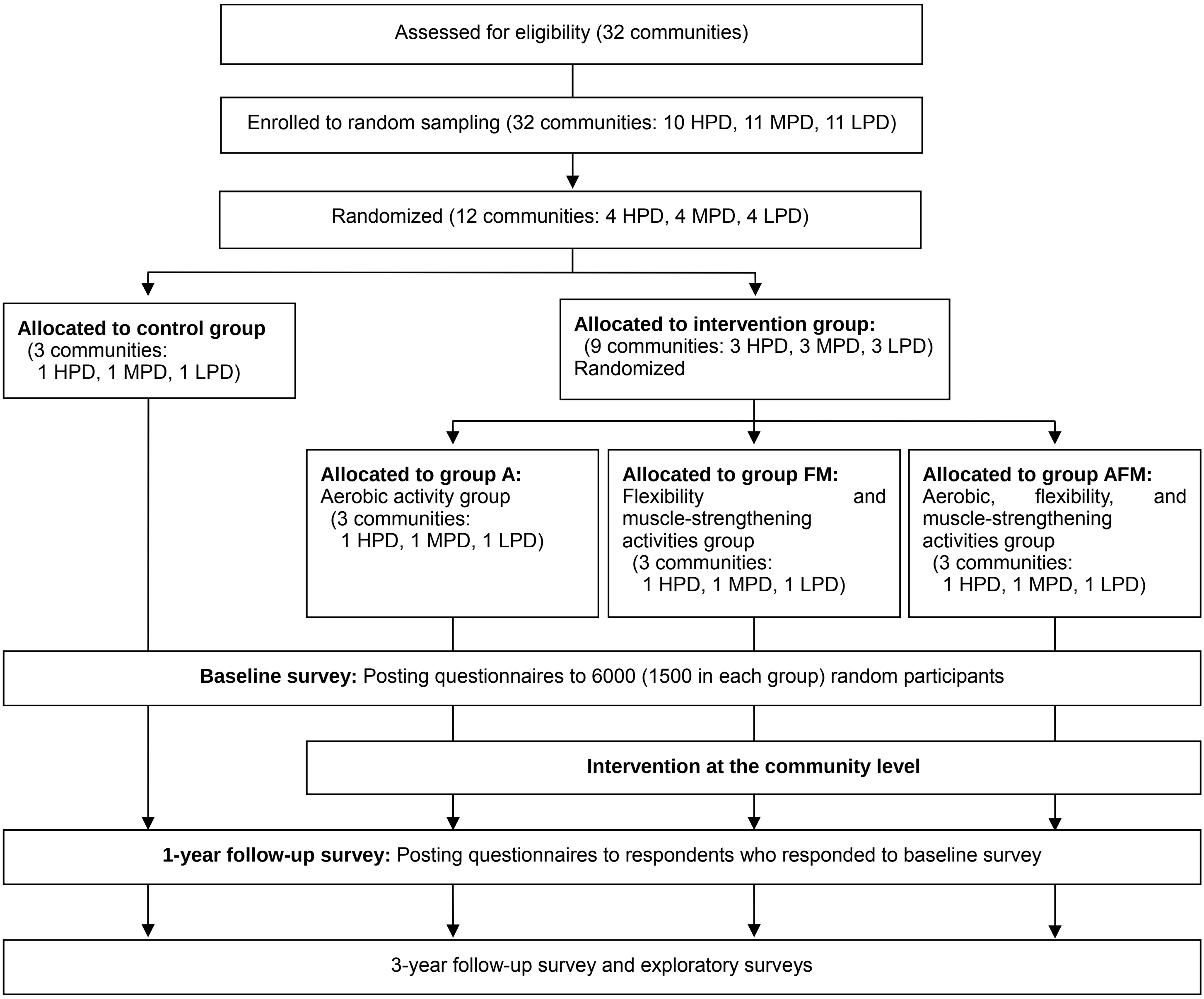

Figure 1: Flow diagram of the trial process

HPD: high population density. MPD: moderate population density. LPD: low population density.

In this study, we have combined flexibility activity with muscle-strengthening activity into Group FM and named the group “*taisou,*” a Japanese term meaning “general gymnastics.” One reason for doing so is that the term is more familiar to

elderly Japanese people than “stretching and muscle-strengthening activities”, and it is not unusual for them to visualize the two activities as one category. We think the familiarity with the terminology of the delivered messages is essential for an effective CWC. Additionally, whereas walking is usually conducted outside of the home, both flexibility and muscle-strengthening activities are mostly conducted inside the home.[24, 25] Therefore, we think flexibility and muscle-strengthening activities can be combined into one indoor activity group in the CWC.

We did not conduct any other cluster selection process for minimizing the risk of contamination (e.g., geographical distance between individual clusters) other than considering and choosing the unit and size of clusters. Figure 2 shows the location of study areas.

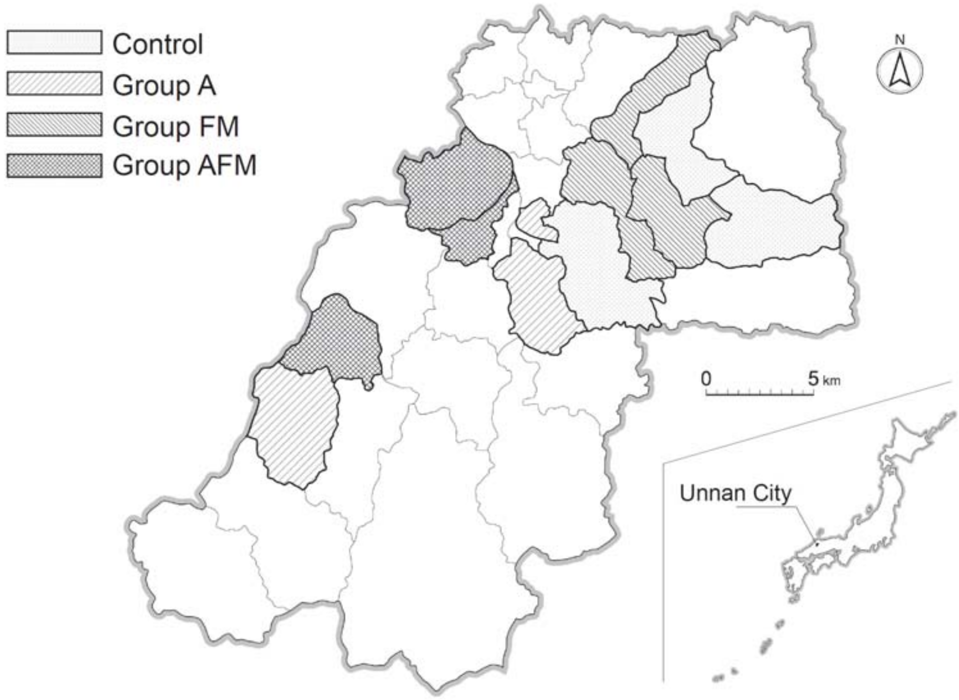

Figure 2: Location of study areas (Unnan, Shimane, Japan)

Group A: aerobic activity group. Group FM: flexibility and muscle-strengthening activities group. Group AFM: aerobic, flexibility, and muscle-strengthening activities group.

4. Intervention

A CWC is to be presented at the cluster level within the intervention groups for a one-year period. In all intervention groups the key message of "*be active*" will be delivered to middle-aged and elderly people (40–79 years) living in the communities, and all groups (A, FM, and AFM) will focus on promoting specific activities. In Group A, mainly walking behavior is to be promoted for aerobic activity. In Group FM, mainly stretching exercises for back muscles, adductor muscles, gluteus maximus, knee extensor muscles (quadriceps), and knee flexor muscles (hamstrings), as well as muscle-strengthening activities for trunk flexor, knee extensor (quadriceps), and knee flexor (hamstrings) are to be promoted. These anatomical areas were chosen as key muscle groups for treating low back and knee pain, which are common, and do not require expensive training equipment.[17, 26, 27] In Group AFM, all of these walking, stretching, and muscle-strengthening activities are to be promoted.

4.1. Social marketing

Social marketing is the use of marketing principles and techniques to influence a target audience to voluntarily accept, reject, modify, or abandon a behavior for the benefit of individuals, groups, or society as a whole.[28] It has been shown to be effective in changing behaviors, and there are several online platforms for health professionals to use for carrying out social marketing and health campaigns.[29-31] We adopted the following social marketing campaign implementation program:

(1) Situational analysis. A situational analysis helps health professionals understand factors that may influence a health campaign and provides background and context to the social marketing plan. We conducted the following situational analyses: background and issue identification, environmental scan, and SWOT (Strengths, Weaknesses, Opportunities, Threats) analysis.[31]

(2) Market segmentation and targeting. We used the TARPARE model to determine the primary communication target segment.[32] The model assists the health promotion practitioner to systematically compare and select what

might be appropriate target groups when there are a number of segments competing for attention and resources. We placed special emphasis on the total number of persons in the segment (T), at risk status (R), and persuasibility (P) as segment priority factors. In the process of market (population) segmentation, we also adopted the stages of change model.[33] However, there were no data regarding the stages of change in physical activity behavior in this study population, so available behavioral data (i.e., walking times and engagement in stretching) was used to supplement the estimation of segment size. For promotion of aerobic activity, we selected a segment of women, 60–79 years of age, who currently have an interest in but are either not engaged in or are insufficiently engaged in regular walking behavior (less than 150 minutes/week), and who have low back or knee pain, regardless of severity. For flexibility and muscle-strengthening activities, we selected a segment of women, 60–79 years of age, who currently engage in flexibility and/or muscle-strengthening activities, either occasionally or daily, and who have low back or knee pain, regardless of severity. The estimated proportion of the target segment of the 40–79 aged population is 19% for aerobic activities and 16% for flexibility and muscle-strengthening activities, based on data from the Shimane Study in Unnan.[34] This targeting does not mean that our CWC ignores the physical activity in non-target people. Rather we aim that the CWC mainly affects the primary communication target with sophisticated messages and approaches and then it has a ripple effect on non-target people.

(3) Setting objectives. The SMART (Specific, Measurable, Achievable, Realistic, Time-based) objective was set for behavioral change as follows: “To increase the percentage of 40–79 years-old individuals who engage in aerobic, flexibility, and/or muscle-strengthening activities in an intervention group from 58% to 66% over a one-year period.” The baseline percentage was estimated from the available data for Unnan. A previous systematic review reported that the median net increase in the percentage of people who reported being physically active as a result of a CWC was 4.2% (range, -2.9% to 9.4%).[7] We decided on the 8% increase because the area and population sizes are relatively small in this study and the effect of a CWC is potentially greater within the community compared to a larger scale CWC (e.g., state or prefecture level).

(4) Marketing strategy development. A CWC follows the “4 Ps” concept of marketing mix (i.e., making sure the right *Product* is available at the right *Price*, in the right *Place* and is well-*Promoted*). Figure 3 shows the concept of marketing mix and examples of each component for promoting physical activity in this study. For example, when we think of *Product* (i.e., physical activity), the benefits of the product rather than the product itself are emphasized.[35] So rather than promoting physical activity *per se*, campaigns should promote ideas such as feeling good, having increased energy or longevity, according to the identified views of the target segments.[36] In the process of creating messages and materials, formative research is done, including interviews with target and other segments on their lives and values; further, pretesting of materials is done through interviews with the target segment about their feelings toward and impressions of such materials.

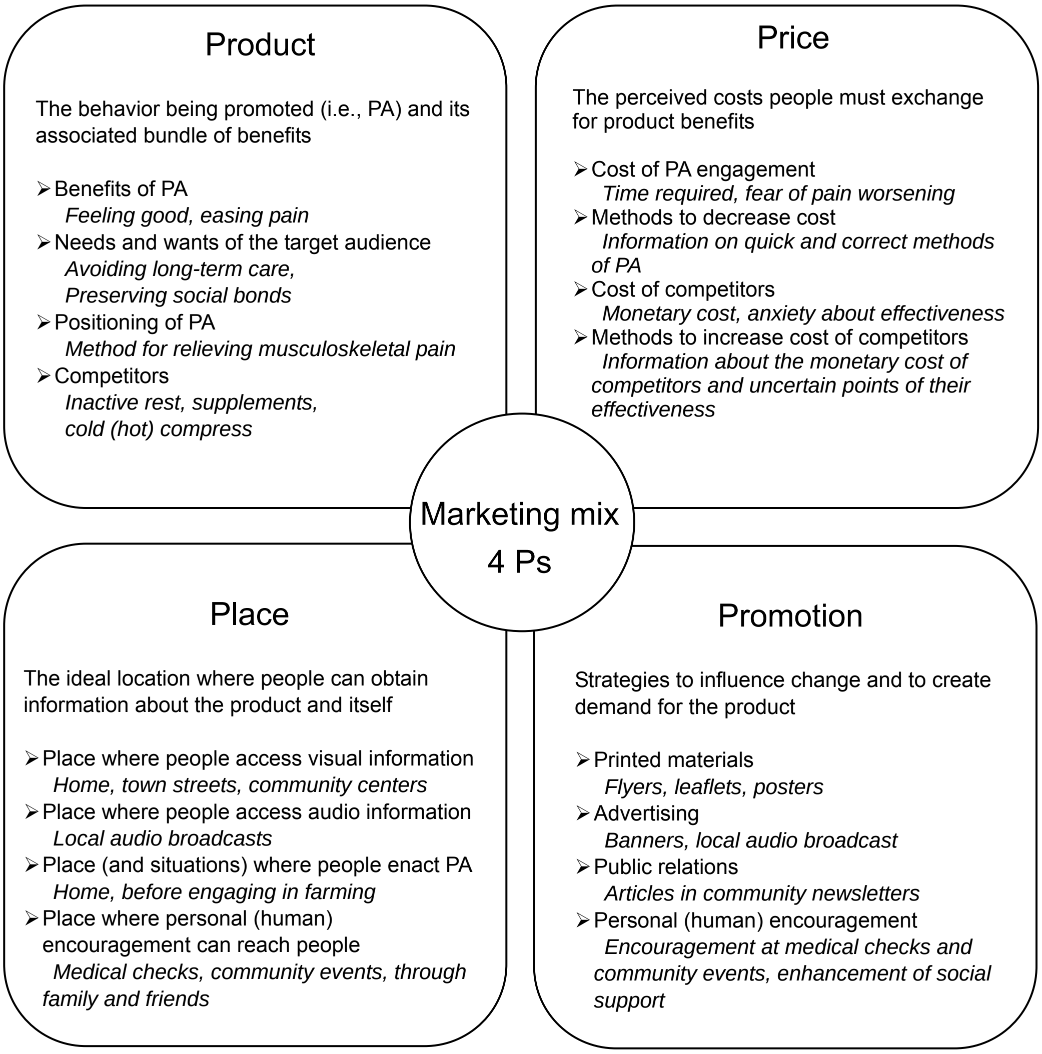

Figure 3: Concept of marketing mix and example elements of the four Ps for promoting physical activity in the COMMUNICATE study
PA: physical activity

After the social marketing process, the key message of “*Be active to cure your low back and knee pain*” (originally in Japanese) was selected, especially for the primary communication target, and is to be delivered within all intervention groups. Acknowledging that the target population experiences low back and knee pain, the intensity and amount of promoted (recommended) aerobic activity are not high, but rather relatively low (e.g., recommendation to start with a daily ten minute walk for current non-walkers), with gradual increases

in intensity and amount. Based on the results of our social marketing overall and formative research regarding flexibility and muscle-strengthening activities, we identified situations in which the local population frequently performed these activities (e.g., before going out, before sleeping, before engaging in farming, after bathing), and we will recommend these in the campaign as specific times and places to perform such activities.

4.2. Campaign component and implementation

A CWC is to be implemented as one of the public health projects of Unnan City Hall. As a consequence of the social marketing process, the CWC consists of three components:

(1) Information delivery: community newsletters, flyers, leaflets, posters, banners, and local audio broadcasts;

(2) Education delivery: outreach health education program and mass - and individual- encouragement by professionals during medical check-ups and various community events, including sports events and festivals;

(3) Support delivery: development of social (peer) support, i.e., promoting encouragement by community leaders and lay health workers; material support, i.e., arranging for residents to get light-reflective materials for walking safety and to buy pedometers[37] (Group A and AFM), and to get video tapes and DVDs on flexibility and muscle-strengthening activities (Group FM and AFM), at each relevant community center; and professional support, i.e., establishing a call center for questions about physical activity and requests for outreach programs.

For cost-effectiveness, the CWC is to be implemented using existing public resources (such as city and community newsletters and local audio broadcasts) which are cost-free, and establishing an outreach program (health education and encouragement) in ongoing community events. A local audio broadcast system is established all over Unnan using a network of cables and can be controlled to broadcast campaign messages limited to specific relevant communities in order to avoid contamination of the intervention. Supplies and costs for producing new materials (leaflets etc.) are to be kept to a minimum, and new independent exercise classes will not be established.

Social marketing, materials production, and the delivery of health education

and encouragement are to be done mainly by a core team consisting of five physical activity professionals, including two researchers, and one public health nurse. Eligibility criteria for the core team physical activity professionals include some form of certification related to physical activity promotion or education, and one or more years of practical physical activity promotion. Criteria for the public health nurse are one or more years in both general public health practice and physical activity promotion in Unnan.

One researcher (MK) is to manage the overall implementation of the campaign and the other (JK) is to assist with management. The other four staff members will manage and coordinate two to three communities (clusters) according to their familiarity with each community. Health education and encouragement will mainly be provided by the staff member managing the particular community, but may also be done by the other core team members.

Workshops on the basic theory and practice of social marketing, including interview skills training, were held for the core team staff. All core team members are aware of the key message of the campaign, and have general knowledge of physical activity recommendations (e.g., recommendations by the American College of Sports Medicine and the American Heart Association[2, 23]) as well as of the benefits and precautions of physical activity for musculoskeletal diseases. The core team is to be trained in brief mass-presentation techniques (delivery an encouraging speech and demonstration of each type of physical activity all within a ten minute period) using a common manuscript to ensure standardization of the intervention. Case report forms of all interventions by the core team will be reviewed by fellow team members. The team will cooperate with the public health (as affiliation), education and sports, and regional development departments of Unnan City Hall, the Unnan Police Department, and with each intervention community's self-administered organization, Senior Citizens' Club, schools and clinics.

For the process assessment of the CWC, qualitative (e.g., content of each leaflet) and quantitative (e.g., number of leaflets) data on each component of the delivered CWC will be recorded. Sample materials used in the CWC are available online (in Japanese).[38] In the three clusters of the control group, no CWC is to be conducted and public health services including health education classes will be delivered by Unnan City Hall in the usual fashion. The usual services provided to the control group, particularly the amount and content of health education, will be recorded.

5. Population-based evaluation

The effectiveness of the intervention will be evaluated using a population-based survey, which will be a prospective cohort design. As a baseline survey, self-administered questionnaires will be mailed to random participants, i.e., those who are randomly sampled using a computer-based resident registry system. Eligible participants will be all men and women aged 40 to 79 years living in the study areas (i.e., 12 communities). Those excluded are individuals in assisted living facilities, those who require long-term care, or those who cannot complete the questionnaires by themselves due to disability. We considered systematically reviewed effective methods to increase response rates,[39] and decided to incorporate the following recommended techniques, i.e., non-monetary incentives, university sponsorship, personalized and relatively short questionnaires, and assurance of confidentiality. We will also send postcard reminders to non-responders, add a preface from the Vice-Mayor to the front cover, make announcements by local audio broadcast and local cablecast, establish a call center for questions, and send a written request for cooperation to community leaders.

Both a one-year and three-year follow-up questionnaire survey from the conducting of the baseline survey is planned (Fig. 1). Follow-up questionnaires will be mailed to the respondents in the baseline survey. Surveys for mortality, functional independence (requirement of long-term care), and morbidity of specific diseases in successive years are also planned as future exploratory studies.

All respondents will give written informed consent to participate in these cohort surveys at the baseline survey. The content of the questionnaires will be the same for all residents. Both participants and data collectors will be randomly-sampled residents. Residents will be blinded to (not informed about) the study design and hypothesis (i.e., the existence of the control group and cluster allocation).[40] While the implementing staff of the CWC (intervention providers) and data analysts will not be blinded to the cluster allocation, the CWC collaborators (e.g., staff of each community’s self-administered organization, Senior Citizens' Clubs, schools and clinics) will be blinded to (not informed about) the study design and hypothesis (the existence of the control group and allocations of other study communities). The Mayor, Vice-Mayor,

supervisory employees, and public health nurses of Unnan City Hall will not be blinded.

6. Measures

6.1. Primary outcome

The primary outcome to be measured is the change in engagement in regular aerobic, flexibility, and/or muscle-strengthening activities from baseline to one-year follow-up. If respondents meet any one of the three following conditions, then they are defined as “engaging in regular physical activity”: (1) engaging in 150 minutes/week or more of walking, (2) engaging in daily flexibility activity, or (3) engaging in muscle-strengthening activities two or more days/week. The threshold of these conditions is based on the physical activity recommendations of the American College of Sports Medicine and the American Heart Association.[2, 23]

Total walking time for both recreation and transport is to be considered as engagement in walking (aerobic activity). Frequency of engagement in flexibility activity will be assessed categorically (daily, not daily but occasionally, not at all). Walking and flexibility items were adopted from the questionnaire used in the Shimane Study. Test-retest reliability (Spearman’s $\rho=0.79$, $P<0.01$) and validity, with average daily step counts recorded by a uniaxial accelerometer, (Spearman’s $\rho=0.41$, $P<0.01$) of walking times in the elderly population were acceptable.[34] The weekly number of days engaged in muscle-strengthening activity is to be assessed using a newly developed questionnaire asking “Do you usually do activities to maintain and/or improve muscles and/or muscle strength (e.g., sit-ups, squats, knee extensions)?” and by identifying the number of days in which they engage in these activities.

6.2. Secondary outcomes

Low back and knee pain are to be evaluated as secondary outcomes. A visual analogue scale (VAS) from 0 mm (no pain) to 100 mm (most intense pain) will be used to assess pain intensity.[41] Additionally, chronic musculoskeletal pain is to be defined as current pain (i.e., an episode of pain during completion of the questionnaire) lasting longer than 3 months within the past 12 months.[42]

We will also evaluate awareness of the campaign for exploratory analysis. Awareness is to be evaluated based on a respondent's encounter with the following CWC components: (1) posters, leaflets, and banners, (2) local audio broadcasts, (3) mass - and individual- encouragement by health professionals during community-based medical check-ups and at community events, (4) individual encouragement by family, friends, and/or neighbors, (5) advice from physicians and medical staff in medical institutions.

Sociodemographic variables will include sex, age, body mass index (BMI) calculated from self-reported weight and height in kg/m^2, self-rated health (excellent; good; fair; poor), years of education, employment status (yes or no), and engagement in farming (yes or no). Most of these variables were considered to be related to physical activity.[43–46] Other information, including overall moderate to vigorous physical activity measured by the short version of the International Physical Activity Questionnaire,[47, 48] pain management, falls, psychological distress,[49] chronic disease history (e.g., heart disease, stroke, diabetes), and general health habits (e.g., smoking, drinking, participation in health education classes) will also be evaluated. Based on the information obtained by the questionnaire, those unable to walk unaided will be excluded from the analyses.

7. Statistical analyses

7.1. Sample size

Based on available physical activity data for Unnan, the estimated rate of participation in regular aerobic, flexibility, and/or muscle-strengthening activities at baseline is 58% with an estimated intracluster (intraclass) correlation coefficient (ICC) of 0.00174. Groups A, FM, AFM, and the control group have equal sample sizes. The primary analysis is a comparison of the three intervention groups with the one control group. We obtained a sample size of 1,536 for the intervention groups and 512 for the control group, using the chi-square test by individual level, imbalanced [3 intervention; 1 control], a two-sided 5% significance level, and a power of 90% to detect an 8% difference in change in engagement in regular physical activity between the intervention and control groups (as described above in the social marketing section), without taking into account the design effect (DE) by the cluster randomization, as

follows:

$$n = \frac{\left(Z_{\alpha} \cdot \sqrt{(1+3) \cdot \frac{p_c + 3p_i}{1+3}\left(1 - \frac{p_c + 3p_i}{1+3}\right)} + Z_{\beta} \cdot \sqrt{3 \cdot p_c(1-p_c) + p_i(1-p_i)} \right)^2}{3 \cdot (p_i - p_c)^2}$$

where

p_i is rate of engagement in regular physical activity in intervention group

p_c is rate of engagement in regular physical activity in control group

To retain power in the cluster randomized trials, the sample size should be multiplied by the DE, which is obtained as follows:[50]

$$DE = 1 + (\overline{m} - 1) \cdot ICC$$

where

DE is design effect

$\overline{m}$ is the average cluster size

ICC is intracluster (intraclass) correlation coefficient

If we plan to recruit an average of 500 participants in each cluster and assume a final sample of 250 in each cluster with a 50% response rate at baseline, the estimated DE is 1.44. The product of the obtained sample size of 512 in the control group and estimated DE of 1.44 is 738. Thus, recruiting nine clusters and 4,500 participants in the intervention groups and three clusters and 1,500 participants in the control group will retain expected statistical power. A total of 6,000 participants in the 12 clusters will be surveyed at baseline.

7.2. Primary and secondary analyses

We will use cluster specific methods because communities rather than individuals (participants) are randomized and the data are to be collected at the individual level. For primary analysis, we will compare the nine intervention clusters to the three control clusters for the primary outcome of engagement in regular aerobic, flexibility, and/or muscle-strengthening activities at one-year follow-up using a generalized linear mixed model (GLMM) with sex, age, BMI,

self-rated health, years of education, employment status, engagement in farming, chronic low back and knee pain, chronic disease history, category of population density of each cluster, engagement in regular aerobic, flexibility, and/or muscle-strengthening activities at baseline, and group allocation (i.e., intervention or control) as fixed effects and community (cluster) where respondents live as a random effect.

As secondary analyses, we will compare the nine intervention clusters to the three control clusters to determine changes in VAS pain scores and chronic pain of low back and knee using a GLMM, adjusted for sex, age, BMI, self-rated health, years of education, employment status, engagement in farming, engagement in regular physical activity at baseline, chronic disease history, and category of population density. In addition to the comparison between all intervention groups and the control group, we will compare each intervention group (i.e., Group A, FM, and AFM) to the control group for primary and secondary outcomes and changes in engagement in each different activity (i.e., aerobic, flexibility, and muscle-strengthening activities) in the same manner using a GLMM.

As exploratory analyses, subgroup analyses will be conducted to detect significant differences in intervention effect, after stratification by sex, age group (40–59 and 60–79), and the market segment (primary communication target segment and the remaining population). Associations for engaging in physical activity, low back and knee pain (VAS scores and chronic pain), awareness of the campaign, and other obtained variables will also be analyzed as exploratory analyses.

We will assign cluster specific mean values to missing values. Significance will be set at $P<0.05$. Analyses will be carried out using SAS version 9.1.3 and SPSS 14.0J.

8. Data management

Data acquisition and management will be conducted by the Physical Education and Medicine Research Center UNNAN.

For the data management, we will use ID numbers to identify the respondents. The information on the questionnaires will be entered into an electronic dataset along with the ID numbers of respondents, rather than their

actual names. The name-ID linkage file will be stored in a locked cabinet separately from the questionnaire dataset.

Regarding data analyses, the basic analyses (e.g., frequency distribution) will be performed by the researchers of the Physical Education and Medicine Research Center UNNAN (MK and JK). Primary and secondary analyses will be performed by the independent statistician (HN).

9. Ethical considerations, safety, and dissemination

As described in Section 5, the effectiveness of the intervention will be evaluated using a population-based survey, which will be a prospective cohort design. All survey respondents will give written informed consent to participate in the cohort surveys at the baseline survey. The intervention will be implemented at community level, and the random-sampled respondents will not necessarily correspond to people who are aware of (or have received) the intervention. Respondents will not be informed about the study design and hypothesis (i.e., the existence of the control group and cluster allocation) and will only be informed about and asked for consent to participate in the cohort design consecutive surveys.

Before implementing the study, the protocol, informed consent, and questionnaires will be reviewed by the research ethics committee of the Physical Education and Medicine Research Center UNNAN. The committee also approves any amendments to the protocol, other than administrative ones. The questionnaire, which includes an informed consent form for the respondent, is appended to the protocol as Section 12.

During the intervention, the implementation core team will keep contact with each intervention community’s self-administered organization, Senior Citizens' Club, schools and clinics. If the team obtains information about negative incidents thought to be caused by the CWC, e.g., a person who gets information about physical activity through the CWC and starts walking suddenly and consequently get worse knee pain, the team will immediately respond and modify the messages of the CWC.

The results of the study will be presented in academic meetings and journals by the study project members when appropriate. Any information concerning an individual that can identify a specific individual will not be presented anywhere.

10. Financial matters

This study is supported by a Grant-in-aid from the Ministry of Health, Labour, and Welfare of Japan (Comprehensive Research on Prevention of Cardiovascular Diseases and Other Lifestyle Related Diseases: H20-Junkankitou-Ippan-001). The funder has no role in the study design, nor will they have a role in the intervention, data collection, analysis, decision to publish, and preparation of the manuscript.

11. References

[1] U.S. Department of Health and Human Services. Physical activity and health: a report of the Surgeon General. Atlanta GA: U.S. Department of Health and Human Services, Centers for Disease Control and Prevention, National Center for Chronic Disease Prevention and Health Promotion; 1996.

[2] Haskell WL, Lee IM, Pate RR, Powell KE, Blair SN, Franklin BA, et al. Physical Activity and Public Health. Updated Recommendation for Adults From the American College of Sports Medicine and the American Heart Association. Circulation. 2007;116:1081-93.

[3] Lee IM, Rexrode KM, Cook NR, Manson JE, Buring JE. Physical activity and coronary heart disease in women: is "no pain, no gain" passe? JAMA. 2001;285:1447-54.

[4] Bauman A, Bull F, Chey T, Craig CL, Ainsworth BE, Sallis JF, et al. The International Prevalence Study on Physical Activity: results from 20 countries. Int J Behav Nutr Phys Act. 2009;6:21.

[5] Guthold R, Ono T, Strong KL, Chatterji S, Morabia A. Worldwide variability in physical inactivity a 51-country survey. Am J Prev Med. 2008;34:486-94.

[6] Task Force on Community Preventive Services. Guide to Community Preventive Services. Campaigns and informational approaches to increase physical activity: community-wide campaigns. Available from: www.thecommunityguide.org/pa/campaigns/community.html, accessed 2009 Oct 1.

[7] Kahn EB, Ramsey LT, Brownson RC, Heath GW, Howze EH, Powell KE, et al. The effectiveness of interventions to increase physical activity. A systematic review. Am J Prev Med. 2002;22:73-107.

[8] Beaudoin CE, Fernandez C, Wall JL, Farley TA. Promoting healthy eating and physical activity short-term effects of a mass media campaign. Am J Prev Med. 2007;32:217-23.

[9] Luepker RV, Murray DM, Jacobs DR, Jr., Mittelmark MB, Bracht N, Carlaw R, et al. Community education for cardiovascular disease prevention: risk factor changes in the Minnesota Heart Health Program. Am J Public Health. 1994;84:1383-93.

[10] Reger-Nash B, Bauman A, Booth-Butterfield S, Cooper L, Smith H, Chey T, et al. Wheeling walks: evaluation of a media-based community intervention. Fam Community Health. 2005;28:64-78.

[11] Reger-Nash B, Bauman A, Cooper L, Chey T, Simon KJ, Brann M, et al. WV Walks: replication with expanded reach. J Phys Act Health. 2008;5:19-27.
[12] Renger R, Steinfelt V, Lazarus S. Assessing the effectiveness of a community-based media campaign targeting physical inactivity. Fam Community Health. 2002;25:18-30.
[13] Tudor-Smith C, Nutbeam D, Moore L, Catford J. Effects of the Heartbeat Wales programme over five years on behavioural risks for cardiovascular disease: quasi-experimental comparison of results from Wales and a matched reference area. BMJ. 1998;316:818-22.
[14] Wray RJ, Jupka K, Ludwig-Bell C. A community-wide media campaign to promote walking in a Missouri town. Prev Chronic Dis. 2005;2:A04.
[15] Young DR, Haskell WL, Taylor CB, Fortmann SP. Effect of community health education on physical activity knowledge, attitudes, and behavior. The Stanford Five-City Project. Am J Epidemiol. 1996;144:264-74.
[16] Hayden JA, van Tulder MW, Tomlinson G. Systematic review: strategies for using exercise therapy to improve outcomes in chronic low back pain. Ann Intern Med. 2005;142:776-85.
[17] American Geriatrics Society Panel on Exercise and Osteoarthritis. Exercise prescription for older adults with osteoarthritis pain: consensus practice recommendations. A supplement to the AGS Clinical Practice Guidelines on the management of chronic pain in older adults. J Am Geriatr Soc. 2001;49:808-23.
[18] Roddy E, Zhang W, Doherty M. Aerobic walking or strengthening exercise for osteoarthritis of the knee? A systematic review. Ann Rheum Dis. 2005;64:544-8.
[19] Zhang W, Moskowitz RW, Nuki G, Abramson S, Altman RD, Arden N, et al. OARSI recommendations for the management of hip and knee osteoarthritis, Part II: OARSI evidence-based, expert consensus guidelines. Osteoarthritis Cartilage. 2008;16:137-62.
[20] Woolf AD, Pfleger B. Burden of major musculoskeletal conditions. Bull World Health Organ. 2003;81:646-56.
[21] Japanese Ministry of Health and Welfare. Comprehensive Survey of Living Conditions of the People on Health and Welfare. 2007. Japanese.
[22] Centers for Disease Control and Prevention. Arthritis as a potential barrier to physical activity among adults with heart disease--United States, 2005 and 2007. MMWR Morb Mortal Wkly Rep. 2009;58:165-9.

[23] Nelson ME, Rejeski WJ, Blair SN, Duncan PW, Judge JO, King AC, et al. Physical Activity and Public Health in Older Adults. Recommendation From the American College of Sports Medicine and the American Heart Association. Circulation. 2007;116:1094-105.
[24] Sasakawa Sports Foundation. The 2008 SSF National Sport-Life Survey. Tokyo: Sasakawa Sports Foundation; 2009. Japanese.
[25] Harada K, Oka K, Shibata A, Ota A, Okada J, Nakamura Y. Factors associated with the stages of change for strength training behavior. Int J Sport Health Sci. 2008;6:251-63.
[26] American Academy of Orthopaedic Surgeons. Treatment of osteoarthritis of the knee (non-arthroplasty). 2008. Available at: http://www.aaos.org/research/guidelines/guidelineoaknee.asp, accessed 2009 Oct 1.
[27] Ettinger WH, Jr., Burns R, Messier SP, Applegate W, Rejeski WJ, Morgan T, et al. A randomized trial comparing aerobic exercise and resistance exercise with a health education program in older adults with knee osteoarthritis. The Fitness Arthritis and Seniors Trial (FAST). JAMA. 1997;277:25-31.
[28] Kotler P, Roberto N, Lee N. Social maketing: improving the quality of life. 2nd ed. Thousand Oaks, CA: Sage; 2002.
[29] National Social Marketing Centre. [Internet]. London: National Social Marketing Cnetre; Available from: http://www.nsmcentre.org.uk/, accessed 2010 Oct 1.
[30] Centers for Disease Control and Prevention. Gateway to Health Communication & Social Marketing Practice. Available from: http://www.cdc.gov/healthcommunication/, accessed 2009 Oct 1.
[31] Health CANADA. Social marketing. Available from: http://www.hc-sc.gc.ca/ahc-asc/activit/marketsoc/index-eng.php, accessed 2009 Oct 1.
[32] Donovan RJ, Egger G, Francas M. TARPARE: a method for selecting target audiences for public health interventions. Aust N Z J Public Health. 1999;23:280-4.
[33] Prochaska JO, DiClemente CC. Stages and processes of self-change of smoking: toward an integrative model of change. J Consult Clin Psychol. 1983;51:390-5.
[34] Kamada M, Kitayuguchi J, Shiwaku K, Inoue S, Okada S, Mutoh Y. Differences in association of walking for recreation and for transport with

maximum walking speed in an elderly Japanese community population. J Phys Act Health. (in press).
[35] Storey JD, Saffitz GB, Rimon JG. Social marketing. In: Glanz K, Rimer BK, Viswanath K, editors. Health behavior and health education: theory, research, and practice. 4th ed. San Francisco, CA.: Jossey-Bass; 2008, p. 435-64.
[36] Cavill N, Bauman A. Changing the way people think about health-enhancing physical activity: do mass media campaigns have a role? J Sports Sci. 2004;22:771-90.
[37] Bravata DM, Smith-Spangler C, Sundaram V, Gienger AL, Lin N, Lewis R, et al. Using pedometers to increase physical activity and improve health: a systematic review. JAMA. 2007;298:2296-304.
[38] Physical Education and Medicine Research Center UNNAN. Research materials. Available from: http://user.kkm.ne.jp/shintai/kankoubutu/kankoubutu-index.html
[39] Edwards PJ, Roberts I, Clarke MJ, Diguiseppi C, Wentz R, Kwan I, et al. Methods to increase response to postal and electronic questionnaires. Cochrane Database Syst Rev. 2009;3:MR000008. DOI: 10.1002/14651858.MR000008.pub4.
[40] Boutron I, Guittet L, Estellat C, Moher D, Hrobjartsson A, Ravaud P. Reporting methods of blinding in randomized trials assessing nonpharmacological treatments. PLoS Med. 2007;4:e61.
[41] Dixon JS, Bird HA. Reproducibility along a 10 cm vertical visual analogue scale. Ann Rheum Dis. 1981;40:87-9.
[42] Wijnhoven HA, de Vet HC, Picavet HS. Explaining sex differences in chronic musculoskeletal pain in a general population. Pain. 2006;124:158-66.
[43] Trost SG, Owen N, Bauman AE, Sallis JF, Brown W. Correlates of adults' participation in physical activity: review and update. Med Sci Sports Exerc. 2002;34:1996-2001.
[44] Sanderson BK, Foushee HR, Bittner V, Cornell CE, Stalker V, Shelton S, et al. Personal, social, and physical environmental correlates of physical activity in rural African-American women in Alabama. Am J Prev Med. 2003;25:30-7.
[45] Sternfeld B, Ainsworth BE, Quesenberry CP. Physical activity patterns in a diverse population of women. Prev Med. 1999;28:313-23.
[46] Kamada M, Kitayuguchi J, Inoue S, Kamioka H, Mutoh Y, Shiwaku K. Environmental correlates of physical activity in driving and non-driving rural Japanese women. Prev Med. 2009;49:490-6.

[47] Craig CL, Marshall AL, Sjostrom M, Bauman AE, Booth ML, Ainsworth BE, et al. International physical activity questionnaire: 12-country reliability and validity. Med Sci Sports Exerc. 2003;35:1381-95.
[48] Murase N, Katsumura T, Ueda C, Inoue S, Shimomitsu T. Reliability and validity study of the Japanese version of the International Physical Activity Questionnaire(IPAQ). Journal of Health and Welfare Statistics. 2002;49:1-9. Japanese.
[49] Hamano T, Yamasaki M, Fujisawa Y, Ito K, Nabika T, Shiwaku K. Social capital and psychological distress of elderly in Japanese rural communities. Stress Health. Epub 2010 Jun 28.
[50] Campbell MK, Elbourne DR, Altman DG. CONSORT statement: extension to cluster randomised trials. BMJ. 2004;328:702-8.

「運動と健康に関する調査」ご協力のお願い

日頃は、市の保健事業につきまして、ご理解とご協力を賜り、厚くお礼申し上げます。

さて、雲南市では、平成 18 年 4 月より「身体教育医学研究所うんなん」を設立し、市民の皆様が生涯健康でいきいきと生活が送れますよう、健康に関する様々な実践活動や調査・研究を進めております。

この度、その一環として「運動と健康に関する調査」を行うことといたしました。この調査では、島根大学医学部環境予防医学教室の支援のもと、雲南市に在住されている 40～79 歳の方の中から地域・年代別に対象者を無作為に選び、運動習慣や健康の状態を調査させていただくものです。対象として選ばれた方には、今年度から継続して（21、22、24 年度）アンケートを郵送させていただきます。

調査データは統計的に処理されるため、ご回答いただいた方が特定されるような形で外部に出ることは決してありません。雲南市の個人情報保護条例を遵守し、データは厳重に管理いたします。なお、調査結果につきましては、今後の市政に活かすとともに、広報やホームページ等を通して公表させていただきます。

つきましては、ご理解の上、調査へのご協力をお願い申し上げます。

☆ 調査票に回答・ご署名の後、<u>同封の返信用封筒に入れて 11 月　日まで</u>に投函（切手不要）をお願いいたします。
ご回答をもって調査への承諾とさせていただきます。

平成 21 年 11 月　　日
雲南市　健康福祉部
身体教育医学研究所うんなん
所長　藤井　勤　（雲南市副市長）

＜お問い合わせ＞
雲南市　健康福祉部
身体教育医学研究所うんなん
電話：0854-45-0300

「身体教育医学研究所うんなん」の活動につきましては、雲南市の市報、ホームページにてご紹介しております。URL：http://www.city.unnan.shimane.jp/

※同封の返信用封筒に入れて
11月　　日　までに投函
（切手不要）をお願いいたします。

運動と健康に関する調査
アンケート調査票

このアンケートには、継続して調査させていただくために、どなたのご回答かが分かるようお名前を書いたシールを貼っています。お答えいただいた内容に関するデータは厳重に管理し、また、調査データは統計的に処理されるため、ご回答いただいた方が特定されるような形で外部に出ることは決してありませんので、安心してお答え下さい。

このページの下からアンケートがはじまります。

- 回答に正解、不正解はありません。あなたのお考えで率直にご回答下さい。
- 同じような質問が繰り返されることがありますが、いずれも大切な質問ですのでご回答下さい。
- 意味の分かりにくい質問があるかもしれませんが、飛ばさず回答として最も近いものをお選び下さい。

ご協力のほど、よろしくお願い申し上げます。

調査責任者
雲南市健康福祉部　身体教育医学研究所うんなん
電話：0854-45-0300

◆各質問について、数字や文字を記入したり、あてはまる番号を〇でかこんだりしてください。

1. 現在のあなたの健康状態はいかがですか？

1．とてもよい　　2．まあよい　　3．あまりよくない　　4．よくない

2. 現在、運転免許（原付、普通二輪、普通、大型など）をお持ちですか？

1．持っていて、よく運転する　　2．持っているが、ほとんど運転しない　　3．持っていない

3. ペット（犬など）の散歩に行くことがありますか？　　1．はい　　2.いいえ

4. ここ一ヶ月の農作業時間はどのくらいですか？
※していない場合は「0」を記入（以下同様）

週　[ ]　日、１日　[ ]　時間　[ ]　分

５. 身長、体重　　　　身長［　　］cm　　体重［　　］kg

６. ふだんの外出頻度（仕事（農作業も含める）、買い物、通院などで家の外に出る頻度）はどれくらいですか？ 庭先のみやゴミ出し程度の外出は含みません。

1. 毎日1回以上	2. 2～3日に1回程度
3. 1週間に1回程度	4. ほとんど外出しない（1週間に1回未満）

７. 友だち・近所の人あるいは別居家族や親戚と会っておしゃべりする頻度はどれくらいですか？

1. 週に２日以上	2. １週間に１回程度
3. １か月に１回程度	4. ほとんどない

８. 外出するにあたってどなたかの介助が必要ですか　　1. 必要　2. 必要ない

９. 趣味や運動としてのｳｫｰｷﾝｸﾞや散歩（犬の散歩含）をどのくらいしていますか？

週［　］日、１日［　　］分

10. 普段、外出（通勤、通院、畑や商店・スーパーまでの移動、集会所や近所への用事など）で 10 分以上継続して歩くことはありますか？（買い物中などのぶらぶら歩行、９. で答えたｳｫｰｷﾝｸﾞなどは除く）

週［　］日、１日［　　］分

11. ストレッチング（筋（すじ）伸ばし・柔軟体操）など、からだを伸ばしたりほぐしたりすることがありますか？

1. １日に１回以上はする　2. 毎日ではないがたまに　3. ほとんどしない

12. 普段、筋力トレーニングなど、筋力（筋肉）を保ったり、高めたりする運動をすることがありますか？　※腹筋運動、スクワット、脚の曲げ伸ばし運動などを含む

週［　］日

13. その他、<u>月１回以上</u>されている運動があれば、○をつけて下さい。（いくつでも）　　0. なし

1. ジョギング・ランニング	2. サイクリング	3. 水泳、水中運動	4. テニス
5. 卓球	6. バドミントン	7. ゴルフ（コース・練習場）	8. ゲートボール
9. グラウンドゴルフ	10. 社交ダンス	11. 太極拳	12. ヨガ
13. バレーボール	14. ボウリング	15. その他（　　　）	

以下（14～19）の質問内容は、みなさんがどの程度の時間、身体を動かしているかについてです。回答にあたっては以下の点にご注意下さい。

- ◆ **強い身体活動**とは、身体的にきついと感じるような、かなり呼吸が乱れるような活動を意味します。
- ◆ **中等度の身体活動**とは、身体的にやや負荷がかかり、少し息がはずむような活動を意味します。

以下（14～19）の質問では、**1回につき少なくとも10分間以上続けて**行う身体活動について**のみ**考えて、お答えください。

※先ほどの質問と似た内容ですが、活動の強度別にお尋ねしていますので、改めてお答え下さい。

14. 平均的な1週間では、**強い**身体活動（重い荷物の運搬、自転車で坂道を上ること、ジョギング、テニスのシングルスなど）を行う日は何日ありますか？　週 ☐ 日

15. **強い**身体活動を行う日は、通常、1日合計してどのくらいの時間そのよう活動を行いますか？　1日 ☐ 時間 ☐☐ 分
※14で「0」と答えた場合「0」と記入

16. 平均的な1週間では、**中等度の**身体活動（軽い荷物の運搬、子どもとの鬼ごっこ、ゆっくり泳ぐこと、テニスのダブルス、カートを使わないゴルフなど）を行う日は何日ありますか？**歩行やウォーキングは含めないで**お答え下さい。　週 ☐ 日

17. **中等度の**身体活動を行う日には、通常、1日合計してどのくらいの時間そのような活動を行いますか？　1日 ☐ 時間 ☐☐ 分
※16で「0」と答えた場合「0」と記入

18. 平均的な1週間では、10分間以上続けて**歩く**日は何日ありますか？ここで**歩く**とは**仕事**や**日常生活**で歩くこと、ある場所からある場所へ**移動**すること、あるいは趣味や運動としての**ウォーキング**、**散歩**など、全てを含みます。　週 ☐ 日

19. そのような日には、通常、1日合計してどのくらいの時間歩きますか？　※18で「0」と答えた場合、「0」と記入。　1日 ☐ 時間 ☐☐ 分

20. の質問は、毎日**座ったり寝転んだりして**過ごしている時間（仕事中、自宅で、勉強中、余暇時間など）についてです。すなわち、机に向かったり、友人とおしゃべりをしたり、読書をしたり、座ったり、寝ころんでテレビを見たり、といった全ての時間を含みます。なお、睡眠時間は**含めない**で下さい。

20. **平日には**、通常、1日合計してどのくらいの時間**座ったり寝転んだりして**過ごしますか？

1 日 ［　　］時間 ［　　］分

＜肩こり（肩のはりや痛み含）についてお聞きします。＞

21. これまでに、肩こりの経験がありますか？

1. ない　2. 右肩だけ　3. 左肩だけ　4. 両肩

22. これまでに、医師の診察が必要になるような肩のケガをした経験がありますか？

1. ない　2. 右肩だけ　3. 左肩だけ　4. 両肩

23. **この1年間**、肩がこることがありましたか？

1. ない　2. 右肩だけ　3. 左肩だけ　4. 両肩

⇒あった方にお尋ねします。その肩こりはどのくらい続きましたか（続いていますか）？

1. 7日未満　2. 1～4週間　3. 1ヶ月以上3ヶ月未満　4. 3ヶ月以上

⇒その肩こりは、**現在も**続いていますか？

1. はい　2. いいえ

24. この 1 年間、肩こりのために、受診することがありましたか？

1. なし　2. 1回　3. 2～6回　4. 7回以上

25. 次の線は、**この数日間（ここ 2、3 日）の肩こりの程度**をおたずねするものです。

左の端を「肩こりなし」、右の端を「最も激しい肩こり」としたときに、この数日間のあなたの肩こりの程度はどのあたりでしょうか？

線の上でこのあたりと思われるところに×印をつけてください。

|―――――――――――――――――――――|

肩こりなし　　　　　　　　これまでに経験した最も激しい肩こり

※記入例

|――――――――×――――――――――――|

肩こりなし　　　　　　　　これまでに経験した最も激しい肩こり

26. 現在、肩こりのために医薬品（飲み薬、湿布等）を使用していますか？使用している場合、何種類か数も書いてください。 ※サプリメントは含みません。

1. はい（______種類） 2. いいえ

＜腰の痛みについてお聞きします。＞

27. これまでに、腰の痛みの経験がありますか？

1. ない 2. ある

28. これまでに、医師の診察が必要になるような腰のケガをした経験がありますか？

1. ない 2. ある

29. この１年間、腰が痛むことがありましたか？

1. ない 2. あった

⇒あった方にお尋ねします。その腰の痛みはどのくらい続きましたか（続いていますか）？

1. 7日未満 2. 1～4週間 3. 1ヶ月以上3ヶ月未満 4. 3ヶ月以上

⇒その腰の痛みは、現在も続いていますか？ 1. はい 2. いいえ

30. この 1 年間、腰の痛みのために、受診することがありましたか？

1. なし 2. 1回 3. 2～6回 4. 7回以上

31. 次の線は、**この数日間（ここ 2、3 日）の腰の痛みの程度**をおたずねするものです。

左の端を「痛みなし」、右の端を「最も激しい痛み」としたときに、この数日間のあなたの痛みの程度はどのあたりでしょうか？

線の上でこのあたりと思われるところに×印をつけてください。

|———————————————|

痛みなし　　　　　　　　　　これまでに経験した最も激しい痛み

32. 現在、腰の痛みのために医薬品（飲み薬、湿布等）を使用していますか？使用している場合、何種類か数も書いてください。 ※サプリメントは含みません。

1. はい（______種類） 2. いいえ

＜ヒザ（膝）の痛みについてお聞きします。＞

33. これまでに、ヒザの痛みの経験がありますか？

1. ない　2. 右ヒザだけ　3. 左ヒザだけ　4. 両ヒザ

34. これまでに、医師の診察が必要になるようなヒザのケガをした経験がありますか？

1. ない　2. 右ヒザだけ　3. 左ヒザだけ　4. 両ヒザ

35. この１年間、ヒザやヒザのまわりが痛むことがありましたか？

1. ない　2. 右ヒザだけ　3. 左ヒザだけ　4. 両ヒザ

⇒あった方にお尋ねします。そのヒザの痛みはどのくらい続きましたか（続いていますか）？

1. 7日未満　2. 1～4週間　3. 1ヶ月以上3ヶ月未満　4. 3ヶ月以上

⇒そのヒザの痛みは、現在も続いていますか？　1. はい　2. いいえ

36. この１年間、ヒザの痛みのために、受診することがありましたか？

1. なし　2. 1回　3. 2～6回　4. 7回以上

37. 次の線は、**この数日間（ここ２、３日）のヒザの痛みの程度**をおたずねするものです。

左の端を「痛みなし」、右の端を「最も激しい痛み」としたときに、この数日間のあなたの痛みの程度はどのあたりでしょうか？

線の上でこのあたりと思われるところに×印をつけてください。

痛みなし　　　　　　　　　　　　これまでに経験した最も激しい痛み

38. 現在、ヒザの痛みのために医薬品（飲み薬、湿布等）を使用していますか？使用している場合、何種類か数も書いてください。　※サプリメントは含みません。

1. はい（______種類）　2. いいえ

39. たばこを吸いますか？

1. 吸う　2. 以前吸っていた　3. 吸わない

→ 1日＿＿＿＿本、　＿＿＿＿歳から＿＿＿＿年間

40. お酒（アルコール飲料）を飲みますか？

1. ほとんど毎日飲む　2. ときどき飲む　3. めったに飲まない

41. 憂鬱（ゆううつ）な気分になることがありますか？

1. はい　2. いいえ

42. 婚姻状況についてお答え下さい。

1. 既婚　2. 未婚

→ 1. 同居　2. 別居　3. 離婚　4. 死別

43. 同居している家族はいますか？

1. はい　2. いいえ

44. 介護が必要な人と同居していますか?

1. はい　2. いいえ

45. 過去1年に転んだことがありますか？「はい」の場合、1年間の転倒回数を記入してください

1. はい（　　回／年）　2. いいえ

→ その転倒でケガや骨折はありましたか？「1. はい」の場合、わかる範囲でその部位を記入して下さい。

1. はい（部位:＿＿＿＿＿＿）　2. いいえ

46. 転倒をすることに対する恐怖感はありますか？

1. 全くない　2. あまりない　3. どちらかといえばある　4. 非常にある

47. 転倒を防ぐために、外出をひかえることがありますか？

1. 全くひかえない　2. ほとんどひかえない　3. 時々ひかえる　4. よくひかえる

48. 小学校入学から最終学歴まで、通算で何年間学校に通いましたか？

（専門学校・大学院は含みますが、予備校や留年・休学は除きます。）

例：中学卒業まで；9年、高校卒まで；12年、

短大・専門学校卒業まで；14年、大卒まで；16年

□ 年間

49. 現在、収入のある仕事のお時間はどのくらいですか？

週 [ ] 日、１日 [ ] 時間

50. あなたは健康に関する教室や講演会、自治会行事、イベント、自主グループ等の活動に参加したことがありますか？あてはまる番号を1つ選んで○をつけて下さい。

1. 週に１回程度　2. 月に1～2回程度　3. 年に数回程度
4. 参加したことはないが参加したいと思う　5. 参加したことはなく関心がない

（折れ線）

51. あなたは、肩・腰・膝などの痛みに対する対処法を見たり聞いたりしたことがありますか？

1. 医師等の専門家から聞いたり、医学専門書等の資料を読んだりしたことがある
2. テレビや雑誌等で見たり、あるいは、専門家ではないが人から聞いたことがある
3. 見たり聞いたりしたことはない

52. これまでに、医師に診断されたことのある疾患があれば、○をつけて下さい。

1. 関節リウマチ　2. 変形性膝関節症　3. 腰部脊柱管狭さく症
4. その他骨運動器疾患（腰痛、膝疾患、足疾患等）　疾患名（　　　　　　）
5. 高血圧症　6. 高脂血症（高コレステロール血症、高中性脂肪血症等）
7. 糖尿病　8. 高尿酸血症・痛風
9. 脳血管障害（脳梗塞、脳出血、くも膜下出血等）
10. 心疾患（心筋梗塞、狭心症、うっ血性心不全等）
11. 腎疾患・泌尿器科疾患（糸球体腎炎、膀胱炎、結石等）
12. 肝臓病（B 型肝炎、C 型肝炎、肝硬変、脂肪肝等）
13. 胃腸疾患（胃炎、胃・十二指腸潰瘍、腸炎等）
14. 内分泌疾患（甲状腺、副腎、脳下垂体等）　15. がん・悪性腫瘍　部位（　　　　　）
16. その他の疾患　病名（　　　　　　）

（折れ線）

以上で全ての質問は終了です。
回答漏れがないかご確認の上、今日の日付とお名前を下の欄にご記入ください。
返信用封筒にてご返送下さいますようお願い申し上げます。

上記の通り回答し、アンケート調査に協力します。

平成 21 年　　　月　　　日

お名前：＿＿＿＿＿＿＿＿

ご協力誠にありがとうございました。
