# Supplementary figures and images for: A community-wide campaign to promote physical activity in middle-aged and elderly people: a cluster randomized controlled trial
Source: Int J Behav Nutr Phys Act. 2013 Apr 9;10:44. doi: 10.1186/1479-5868-10-44 (PMC3637495; doi:10.1186/1479-5868-10-44)

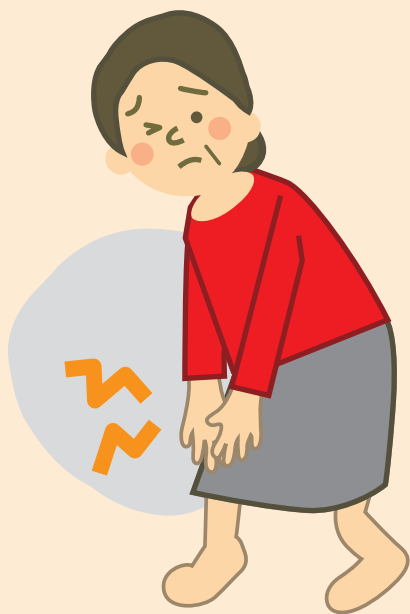

シップに頼る  
だけなんて、  
もうやめた。

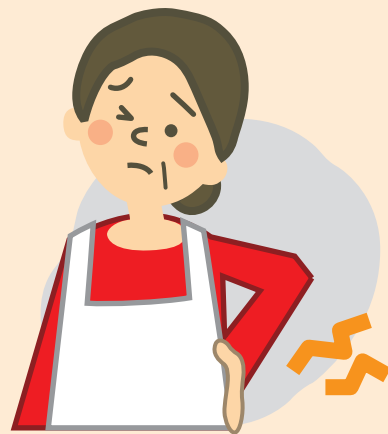

# 腰痛・ひざ痛は 動いて治そう

『腰・ひざ痛に運動キャンペーン中!』

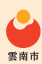

身体教育医学研究所うなん

電話 〇八五四―四五―〇三〇〇

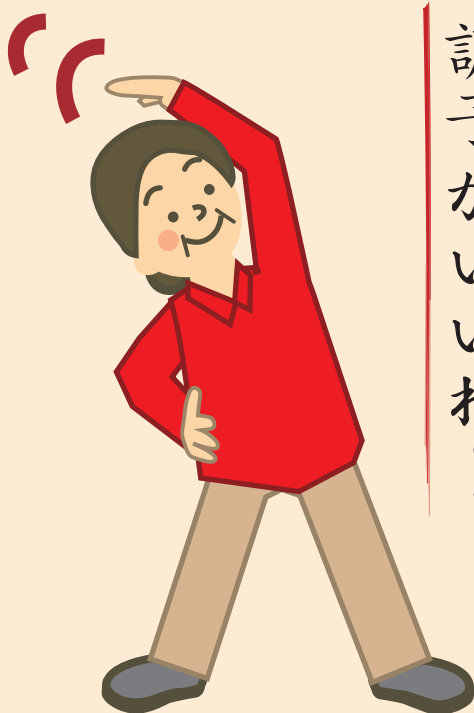

じつとしとるより  
調子がいいわ!

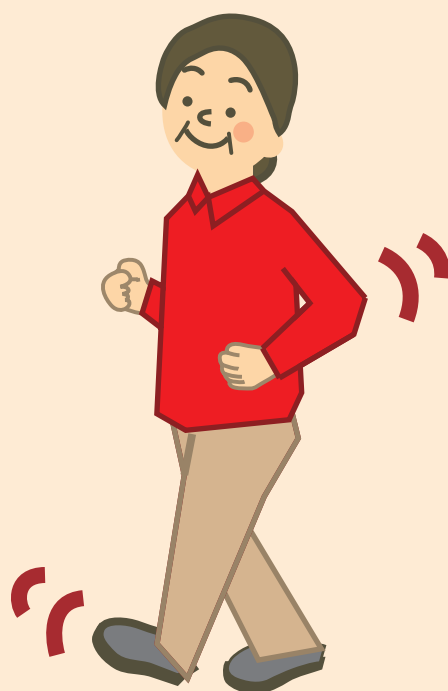

Supplement: Additional file 2 — Poster. Sample material (poster) of the community-wide campaign: COMMUNICATE Study. [file 1479-5868-10-44-S2.pdf]

腰・ひざ痛に運動キャンペーン中！

腰痛・ひざ痛は動いて治そう

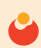

奈良市  
身体教育医学研究所うなん

Supplement: Additional file 3 — Banner. Sample material (banner) of the community-wide campaign: COMMUNICATE Study. [file 1479-5868-10-44-S3.pdf]
